# Supplementary material for: Ocular toxicity, distribution, and shedding of intravitreal AAV-eqIL-10 in horses
Source: Mol Ther Methods Clin Dev. 2024 Oct 28;32(4):101360. doi: 10.1016/j.omtm.2024.101360 (PMC11656199; doi:10.1016/j.omtm.2024.101360)
Supplement: Document S1. Figures S1–S4 and Tables S1 and S2 [file mmc1.pdf]

OMTM, Volume *32*

Supplemental information

Ocular toxicity, distribution, and shedding of intravitreal AAV-eqIL-10 in horses

Kim Young, Tomoko Hasegawa, Naveen Vridhachalam, Nichol Henderson, Jacklyn H. Salmon, Trace F. McCall, Matthew L. Hirsch, and Brian C. Gilger

Table S1. Information on individual horses

Horse	Treatment group	Age	Gender	Breed	Body weight (kg)
1	Vehicle control (V)	11 years	Female	Thoroughbred	507
2	Low dose AAV8-eqIL10 (LD1)	12 years	Female	Pony	413
3	Low dose AAV8-eqIL10 (LD2)	13 years	Male (castrated)	Thoroughbred	569
4	High dose AAV8-eqIL10 (HD1)	20 years	Female	Quarter Horse	399
5	High dose AAV8-eqIL10 (HD2)	22 years	Female	Quarter Horse	499

Table S2. Neutralizing Antibody Testing.

Horse	Serum neutralizing antibodies	
	Prior to injection	Day 84 after injection
V	Not detected	Not detected
LD1	Not detected	Not detected
LD2	Not detected	4
HD1	Not detected	16
HD2	Not detected	16

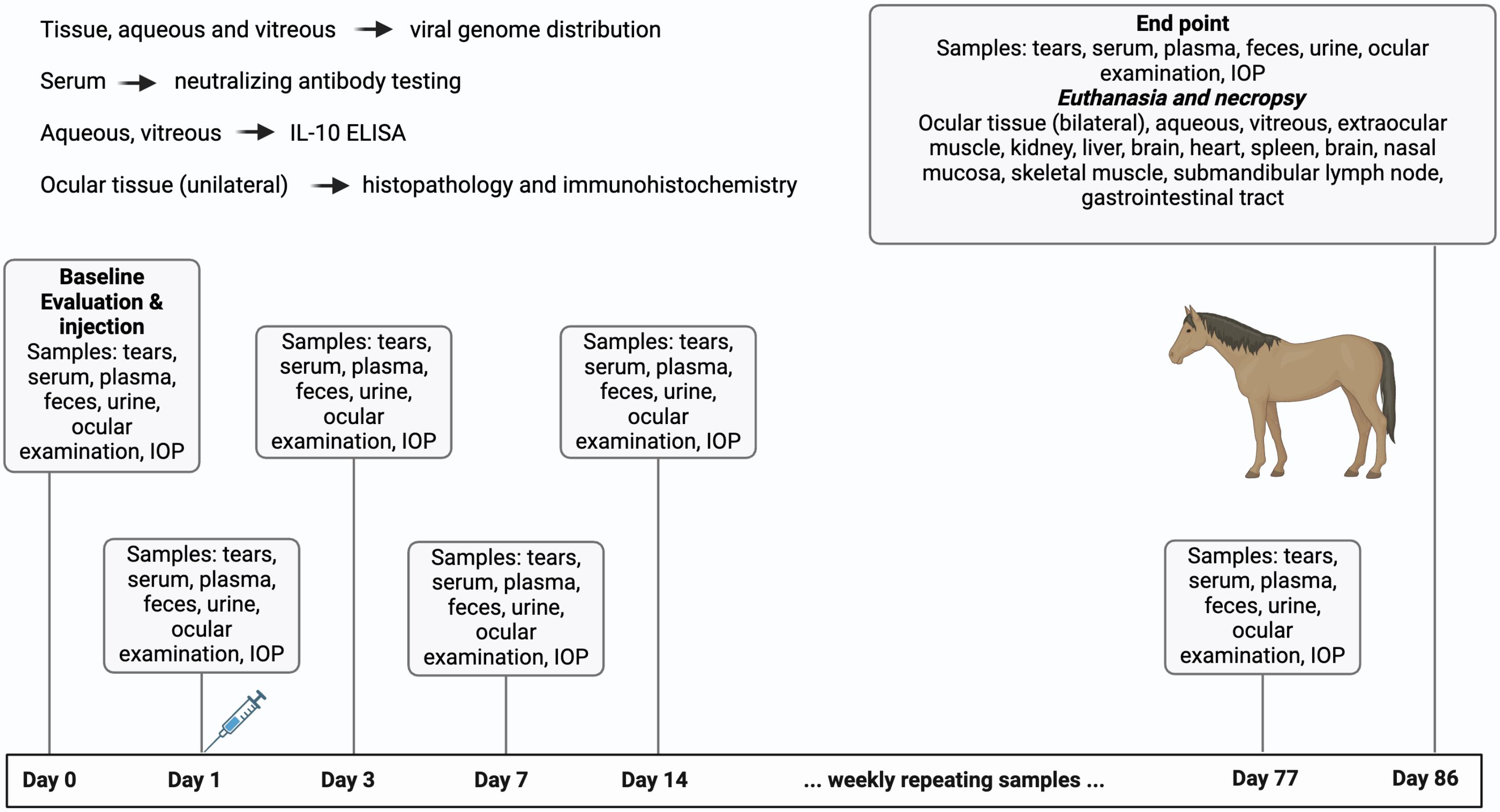

Figure S1. Sampling timeline and endpoint testing

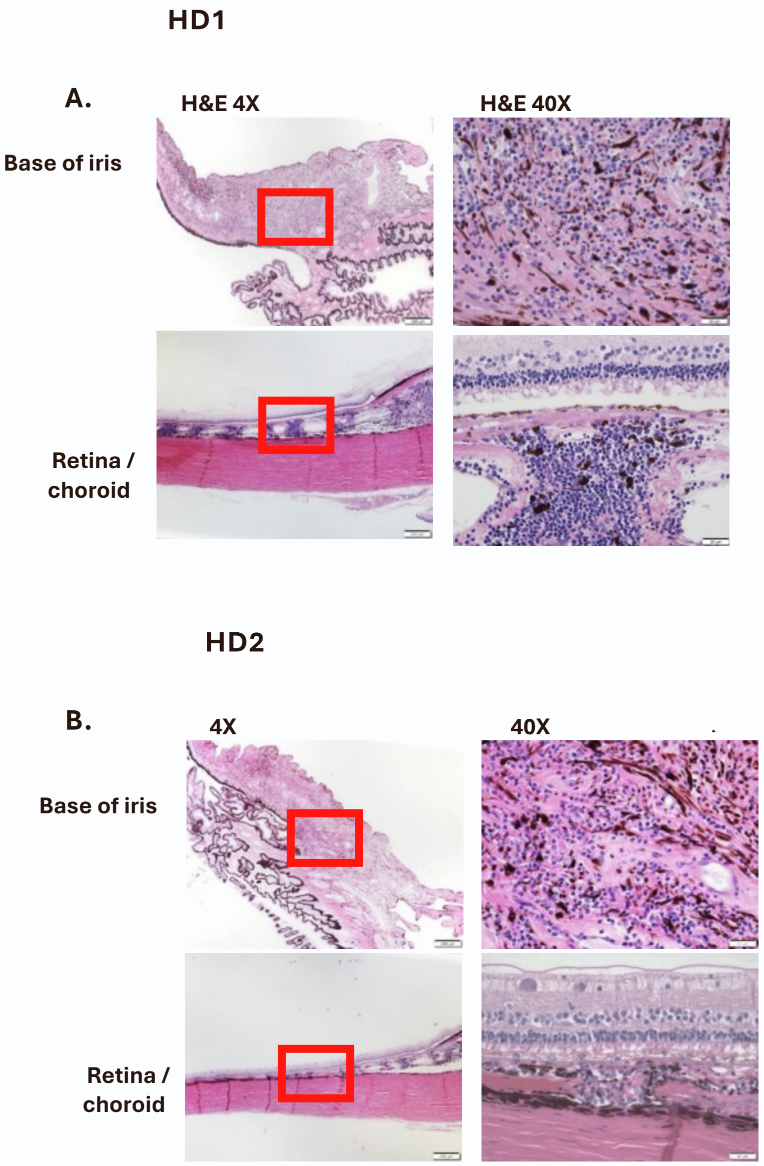

Figure S2 A. HD1. Ocular histology. In the iris and ciliary body (red box), there was diffuse infiltration of mononuclear cells in the iris and ciliary body (top row) and focal areas of mononuclear cells in the choroid (red box). **B.** HD2. Ocular histology. In the iris and ciliary body (red box), there was diffuse infiltration of mononuclear cells in the iris and ciliary body (top row) and focal areas of mononuclear cells in the choroid (red box).

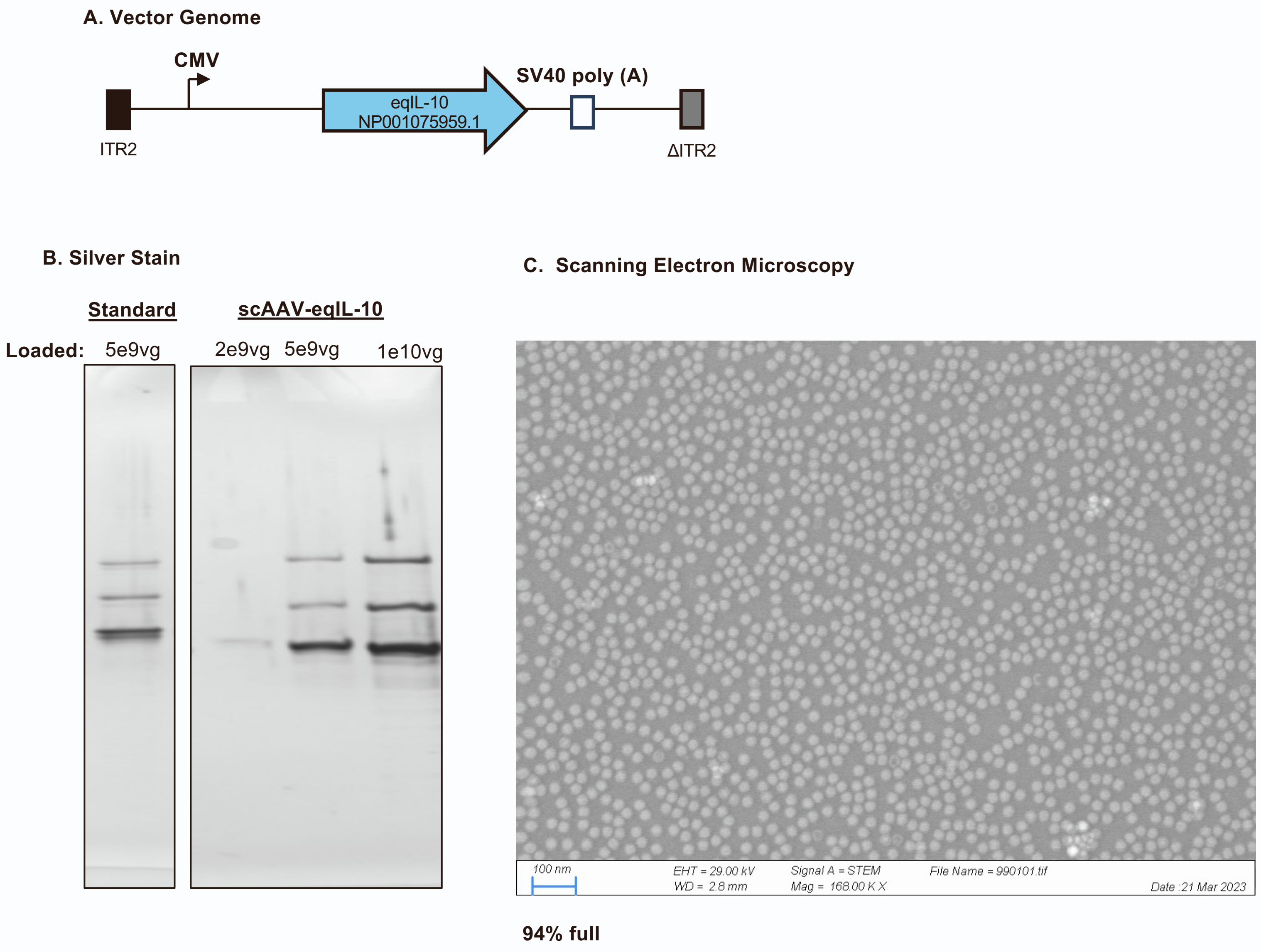

Figure S3. AAV8-eqIL-10 Characterization. **A.** A cartoon of the vector genome is depicted. **B.** Silver stain analysis revealed pure vector preparations at the indicated viral genomes. **C.** The full to empty capsid ratio of the preparation was >90% as demonstrated by electron microscopy.

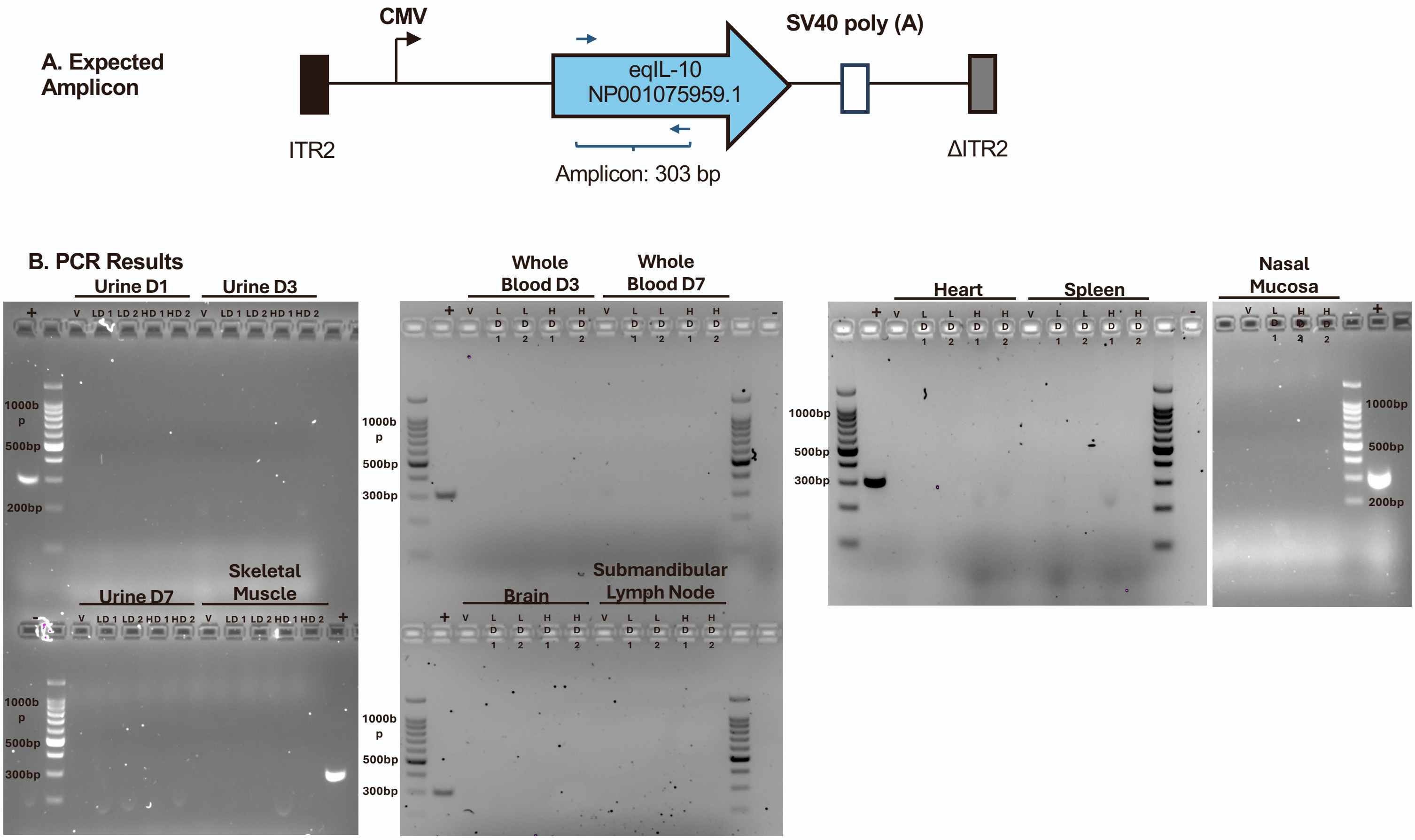

Figure S4. Vector Genome Biodistribution. The distribution of AAV8-eqIL-10 viral genomes in peripheral tissues was investigated via endpoint PCR. **A.** The forward and reverse primers annotated on the vector genome. The expected amplicon was 303 bp. **B.** The PCR amplicons were run on an agarose gel and visualized. All tissues from each horse did not have a 303 bp band, indicating that vector genomes were below the limit of detection in the indicated tissues. + = positive control; - = negative control; D = day; V = vehicle-treated; LD = low-dose treated; HD = high-dose treated.
