# Supplementary material for: Ocular toxicity, distribution, and shedding of intravitreal AAV-eqIL-10 in horses
Source: Mol Ther Methods Clin Dev. 2024 Oct 28;32(4):101360. doi: 10.1016/j.omtm.2024.101360 (PMC11656199; doi:10.1016/j.omtm.2024.101360)
Supplement: Document S2. Article plus supplemental information [file mmc2.pdf]

Molecular Therapy
Methods & Clinical Development
Original Article

Ocular toxicity, distribution, and shedding of intravitreal AAV-eqIL-10 in horses

Kim Young,[1] Tomoko Hasegawa,[2,3] Naveen Vridhachalam,[2,3] Nichol Henderson,[1] Jacklyn H. Salmon,[1] Trace F. McCall,[2] Matthew L. Hirsch,[2,3] and Brian C. Gilger[1]

[1]Clinical Sciences, North Carolina State University, Raleigh, NC 27607, USA; [2]Ophthalmology, The University of North Carolina at Chapel Hill, Chapel Hill, NC 27514, USA; [3]Gene Therapy Center, The University of North Carolina at Chapel Hill, Chapel Hill, NC 27599, USA

Non-infectious uveitis (NIU) is a painful recurrent disease affecting 2%–5% of horses. Current treatments require frequent administration with associated adverse events. In a previous study, intravitreal (IVT) adeno-associated virus (AAV) harboring equine interleukin-10 (eqIL-10) cDNA inhibited experimental uveitis in rats. The goal of this study was to evaluate the ocular tolerability, vector genome (vg) distribution, and vector shedding following an IVT injection of AAV8-eqIL-10 in normal horses with the hypothesis that it would be well tolerated in a dose-dependent manner in horses. Injections were well tolerated with mild transient signs of ocular inflammation; however, horses receiving the highest dose developed keratic precipitates. The vgs were not detected in the tears 3 days after injection, or in urine or feces at any time. Aqueous and vitreous humor eqIL-10 levels increased to higher than 1.5 ng/mL, more than 20 times higher than reported effective endogenous and induced levels. The vgs were detected in ocular tissues, and systemic distribution was identified only in the liver and kidney. No systemic effects were identified 86 days after dosing with IVT AAV-eqIL-10. Further investigation of lower doses of IVT AAV8-eqIL-10 therapy is an important next step toward a safe and effective single-dose treatment of equine uveitis with broader implications for treating NIU in humans.

INTRODUCTION

Uveitis is inflammation of the iris, ciliary body, and choroid. It is associated with both infectious and non-infectious etiologies.[1–3] Non-infectious uveitis (NIU) is a painful disease that is a common cause of blindness in the United States, with 30,000 new cases annually in humans.[4] In horses, a painful, vision-threatening NIU is equine recurrent uveitis (ERU). This disease is characterized by episodes of active ocular inflammation alternating with varying intervals of clinical quiescence and affects an estimated 2%–5% of all horses.[5–8] The pathophysiology and resulting clinical effects of NIU are driven by disruption of the blood-ocular barrier, which normally limits the access of the immune system to intraocular antigens. Any disruption of this barrier can cause the vasculature surrounding the normally privileged region of the eye to become leaky, allowing inflammation-inducing immune cells to enter the eye[9,10] and pathologically react to self-antigens. Clinical uveitis often develops spontaneous recurrent bouts of inflammation, likely from T cells recognizing additional autoantigens in the ocular tissue. This leads to progressive destruction and opacification of ocular tissues, resulting in blindness. In horses, the conventional treatment for ERU is non-specific, including topical and systemic corticosteroids and other topical or oral immunosuppressive agents; however, none are effective in preventing relapses.[5,8] These therapies are also limited by poor treatment compliance and long-term adverse effects, such as corneal degeneration, glaucoma, cataracts, ocular hypertension, and infection, all of which may contribute to the development of blindness.[5,11] Therefore, the development of a long-term and effective therapeutic is important.

Gene therapies are currently in development for several ocular diseases, including inherited retinal degeneration.[12–14] Adeno-associated virus (AAV) vectors are currently the most promising approach, with a relatively impressive safety profile in thousands of treated patients. After a single injection, AAV transduces many cell types and establishes long-term transgene production for years.[15] Different AAV serotypes display altered tropism for ocular cell types and tissues, presumably due to variations in the capsid and host cell receptor affinities, nuclear trafficking, and/or uncoating. AAV serotype 8 (AAV8) has been shown to transduce the ocular uveal tissue effectively and has already been used in clinical applications in the eye.[16–20]

The immunomodulatory cytokine interleukin-10 (IL-10) plays a critical role in limiting host immune responses and preventing autoimmune disorders.[21,22] IL-10 is produced by immune cells and inhibits macrophages, natural killer, T helper 1, and dendritic cell function.[23] IL-10 also inhibits the production of pro-inflammatory cytokines and co-stimulatory molecules and interferes with general antigen-presenting cell function.[21,23,24] IL-10 plays an important role in uveitis by protecting the eye from chronic inflammation and helping to prevent relapses of inflammation. Ocular supplementation of endogenous IL-10 may be a promising therapeutic for ERU and other NIU,

Received 4 May 2024; accepted 24 October 2024;
https://doi.org/10.1016/j.omtm.2024.101360.

Correspondence: Brian C. Gilger, Clinical Sciences, North Carolina State University, Raleigh, NC 27607, USA.
E-mail: bgilger@ncsu.edu

Published by Elsevier Inc. on behalf of The American Society of Gene and Cell Therapy.
This is an open access article under the CC BY license (http://creativecommons.org/licenses/by/4.0/).

and several studies have evaluated the anti-inflammatory effects and efficacy of IL-10 both as a systemic and a localized treatment.[24–27]

Recently, our laboratories demonstrated that AAV-equine IL-10 (AAV-eqIL-10) effectively inhibits uveitis in the well-established experimental autoimmune uveitis (EAU) rat model of NIU.[28] We demonstrated that AAV-eqIL-10 (2.4e−9 to 2.4e−10 vector genomes [vg]) injected intravitreally (IVT) suppressed the development of ocular inflammation in this model. Additionally, vector-derived eqIL-10 cDNA was detected in relevant ocular tissues, such as the iris/ciliary body (ICB) and retina.[28] We also demonstrated that recombinant AAV genomes were inconsistently detected in body organs outside the eye following IVT injection in rats, suggesting limited AAV8 biodistribution in peripheral organs.[29]

Together, these studies demonstrated that the IVT injection of AAV8-eqIL-10 effectively inhibits uveitis, transduces relevant ocular tissues, and poses little off-target risk by having limited systemic biodistribution. Because of the challenge of translating AAV toxicity and biodistribution following IVT injection from rodent eyes to large eyes, such as those of a horse, and to determine toxicity and immune response to ocular administration of AAV vectors in an equine eye, which has not been previously reported, the present study aimed at evaluating the ocular toxicity, distribution, and viral shedding following a single IVT injection of AAV8-eqIL-10 in normal horses.

RESULTS

Dosing

The horses (n = 5) used in this study were donated to North Carolina State University and had physical and ocular examinations and complete blood counts and serum chemistry analysis, all of which were either normal or had clinically insignificant abnormalities. Horses ranged in age from 11 to 22 years, consisted of four females and one male, and represented three horse breeds: Thoroughbreds, Quarter Horses, and pony (Table S1). Horses were maintained on pasture in North Carolina State Laboratory Animal Resources facilities. Five horses were randomly divided into three groups, receiving IVT injections of either balanced saline solution (BSS) (n = 1 horse, two eyes), a low dose (LD) of AAV8-eqIL-10 (3.75×10^{11} vg) (n = 2 horses, four eyes), or high dose (HD) of AAV8-eqIL-10 (3.75×10^{12} vg) (n = 2 horses, four eyes), each in a volume of 500 μL (Figure S1). All injections were bilateral, and all AAV vectors were produced by the University of North Carolina Gene Therapy Center and contained self-complementary genomes.[28] The injections were performed under routine standing intravenous sedation following topical anesthetic (proparacaine HCl) administration. Immediately following injection, ophthalmic examination revealed that the ocular fundus appeared normal without vitreal opacity. No further therapy was required after injection in any of the horses throughout the remainder of the study.

Ocular examination

Ocular examinations (OEs) consisting of slit lamp biomicroscopy, indirect ophthalmoscopy, and tonometry were performed on days 0, 1, 3, and 7 and weekly until day 86 post-IVT injection (Figure S1). Mild conjunctival hyperemia and chemosis were observed on days 1 and 3 after injection in one of the vehicle-dosed eyes, three of the LD-dosed eyes, and two of the HD-dosed eyes, but these findings resolved in all eyes by day 7 after injection (Figure 1; Table 1). The right eye of an LD horse (LD 2) had aqueous flare (1+) and conjunctival hyperemia from days 28–49, which resolved spontaneously, while one HD horse (HD 2) had mild conjunctival hyperemia in the left eye on day 35 after injection. One horse in the HD group (HD 2) also developed peripapillary depigmentation in the right eye on day 21 after injection, which did not change throughout the remainder of the study period (Figure 2; Table 1). Both horses in the HD group developed bilateral keratic precipitates (KP) on days 70 and 77, respectively (Figure 2; Table 1), which persisted until the end of the study. Additionally, mean intraocular pressure (IOP) was significantly lower in HD eyes compared to LD and BSS eyes on days 28–86 (Figure 1).

Vector shedding

Tear, urine, and feces samples from each horse were analyzed for vector shedding via PCR/qPCR of the transgenic cDNA. No vgs were detected in urine or feces before or after injection at any time point (Table 2). Tear samples were negative before injection, but on day 1 after injection, both LD horses had eqIL-10 genome detection in tears from both eyes (in four of four eyes), while tears from one eye of one HD horse were positive (in one of four eyes). On day 3, one LD horse had detection in one eye (in one of four eyes). All samples were found negative at day 14 post-injection (Table 2).

eqIL-10 ELISA

Concentrations of eqIL-10 were measured in the aqueous (AH) and vitreous humor (VH) by ELISA sampled at the time of euthanasia on day 86 after injection. In the AH, there was a dose-related increase in eqIL-10 ranging from a mean of 0.2 ng/mL in the vehicle eyes and a mean of 1.9 ng/mL in the LD eyes to a mean of 4.6 ng/mL in the HD eyes (Figure 3). In the VH, the eqIL-10 concentrations were less dose related, with the vehicle-dosed eyes having a mean of 0.1 ng/mL, the LD eyes having a mean of 6.8 ng/mL, and the HD eyes with a mean concentration of 6.9 ng/mL of eqIL-10. The eqIL-10 concentrations in LD and HD eyes were significantly higher than the vehicle eqIL-10 concentration ($p = 0.04$; two-way ANOVA), but the eqIL-10 LD and HD concentrations were not significantly different (Figure 3). It should be noted that there was variability between subjects, but not within, regarding the amount of eqIL-10 produced in the vitreous of the LD group.

Histological findings

There were no abnormal histologic findings in the eyes dosed with BSS vehicle. In eyes dosed with LD of AAV8-eqIL-10, slight, diffuse lymphocytic-plasmocytic (LP) cellular infiltrate was present in the iris, ciliary body, and pars plana. There were also small focal areas of LP cells in the choroid inferiorly, but the retina and other ocular tissues appeared normal (Figure 4). In the HD group, both eyes examined had diffuse moderate LP cellular infiltration into the base of the iris, the ciliary body processes, and pars plana. There were inferior multifocal areas of LP cellular infiltrate in the choroid. The retina and other ocular tissues appeared normal (Figures 4 and S2).

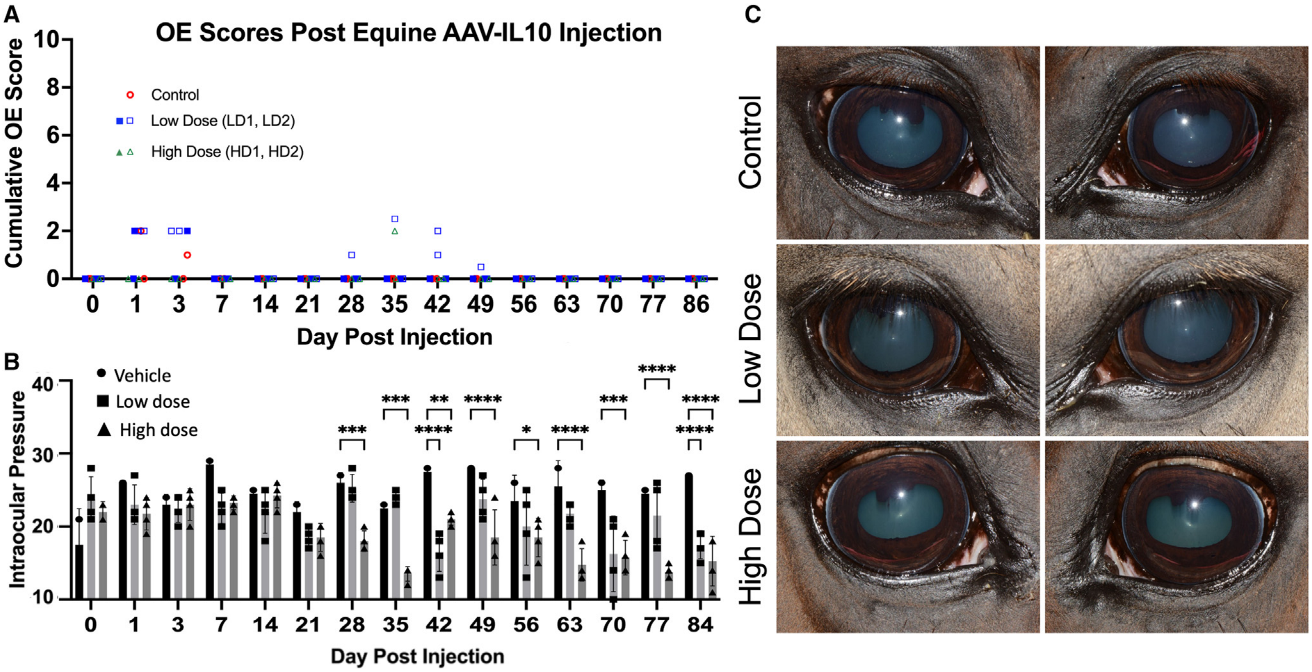

Figure 1. Clinical examination images

(A) Cumulative inflammatory scores of ocular examinations revealed only mild post-injection signs of mild inflammation for the first days after injection and in LD (☐) horse 2 from days 28–49 and one HD horse 2 (Δ HD) at day 35 after dosing. (B) Mean intraocular pressures (IOPs) of the LD and HD eyes were significantly lower than the control saline-dosed eyes at variable time points from 28 to 84 days after injection. Data represented as mean ± SEM. *$p < 0.05$; **$p < 0.01$; ***$p < 0.001$; ****$p < 0.0001$; ANOVA with Tukey's. (C) Representative clinical photographs of eyes at 63 days after injection.

Ocular distribution

AAV8 vgs were quantified via qPCR in multiple ocular tissues, including the conjunctiva, cornea, ICB, choroid, retina, optic nerve, and the AH and VH. The vgs were detected in most ocular tissues sampled from eyes dosed with either LD or HD AAV8-eqIL-10, but vgs were not detected in the vehicle-dosed eyes (Figure 5). No vgs were detected in the optic nerve for LD- or HD-treated eyes. In both LD and one HD, the retina (LD average = 1.3×10^3 vg/ng DNA; HD average = 8.85×10^2 vg/ng) and ICB (LD average = 6.63×10^2 vg/ng; HD average = 3.65×10^2 vg/ng) contained the highest overall quantity of average vg/ng of genomic DNA (gDNA) (Figure 5). One HD subject experienced a comparatively higher quantity of vgs in the choroid compared to eyes from the other treatment groups. A lower but quantifiable number of vgs were also detected in the cornea of both LD horses and the conjunctiva of all but one LD horse (Figure 5). VH and AH data were normalized to the volume of the collected solution. In all AAV-treated horses, vgs were detected in both the VH and the AH. The vitreous contained the highest quantity of vg/μL of solution (LD average = 1.15×10^5 vg/μL; HD average = 4.22×10^5 vg/μL), while the AH was found to contain a comparatively lower quantity of vg (LD average = 1.40×10^4 vg/μL; HD average = 5.17×10^4 vg/μL). These data indicate a similar ∼3.7-fold difference in quantified vgs between LD and HD eyes in both AH and VH samples (Figure 5).

Peripheral organ distribution

To determine the distribution of vgs among the peripheral organs following IVT injection, total DNA was harvested from various tissues and analyzed for transgenic eqIL-10 amplification by endpoint PCR and/or qPCR. The vgs were detected at a low but quantifiable concentration in the liver of one LD horse and both HD horses and the kidney of a single HD horse (Figure 5; Table 3). The vgs were not detected in any other peripheral organs examined, including the heart, spleen, brain, nasal mucosa, skeletal muscle, submandibular lymph node, gastrointestinal (GI) tract, and whole blood (days 3 and 7 after injection) for any horse.

Neutralizing antibody

Before AAV8-eqIL-10 administration, the serum from all subjects was negative for AAV8-neutralizing antibodies (Table S2). Serum collected 86 days after injection exhibited low levels of AAV8 neutralizing antibodies, with one LD horse (LD 2) having an antibody titer of 1–4 serum dilution and the two HD horses having an antibody titer of 1–16 serum dilution. The horse dosed with BSS was negative (Table S2).

DISCUSSION

Recurrent uveitis is a painful and vision-threatening disease that impacts an estimated 2%–5% of the equine population.[5,6,8] Its chronicity and complex pathophysiology require the use of long-term therapies that are prone to compliance failures and drug-related ocular

Table 1. Clinical adverse events observations

Horse	Dose	Adverse event	Days identified[a]
1. Saline control	not applicable	none	not applicable
2. LD AAV-eqIL-10 (LD 1)	3.75e−11 vgs	none	not applicable
3. LD AAV-eqIL-10 (LD 2)	3.75e−11 vgs	conjunctival hyperemia OD aqueous flare (scores 0.5–1)[b] OD	35–49
4. HD AAV-eqIL-10 (HD 1)	3.75e−12 vgs	keratic precipitates OU	77–86
5. HD AAV-eqIL-10 (HD 2)	3.75e−12 vgs	peripapillary depigmentation OD<br>conjunctival hyperemia OS<br>keratic precipitates OU	21–86<br>35<br>70–86

OD, right eye; OS, left eye; OU, both eyes; vg, viral genome.
[a]Ocular examinations were performed on days 0, 1, 3, and 7, and then weekly until day 86.
[b]Modified SPOTs (semiquantitative preclinical ocular toxicology) scoring system was used to generate numerical scores.

complications, including corneal degeneration, secondary infections, glaucoma, and cataracts.[5] Furthermore, systemic administration of immunosuppressants, analgesics, and corticosteroids poses risks to horses, including insulin dysregulation, laminitis, and colic.[30–32] Current therapy deficiencies result in many affected horses losing their eyes (∼12%), resulting in a decreased monetary value and possible euthanasia because of poor disease control (∼15%).[5] Therefore, a safe, effective, long-term treatment for recurrent uveitis is needed.

Immunomodulation using IL-10 has been identified as a possible therapeutic in many diseases, including Crohn disease, rheumatoid arthritis, psoriasis, and other immune-mediated syndromes.[33] Recently, AAV-mediated expression of IL-10 has been evaluated in a canine model of ventricular arrhythmia,[34] and it has also been shown to decrease inflammatory mediators after intra-articular dosing in an equine inflammatory joint model.[35] Our laboratories demonstrated that a single IVT injection of AAV8-eqIL-10 effectively inhibited EAU in a rat model.[28] However, before considering this promising therapeutic for treating equine uveitis in clinical patients, we needed to demonstrate the tolerability, safety, toxicity, and shedding in normal horses.

This study tested the hypothesis that IVT AAV8-eqIL-10 gene therapy would be well tolerated in a dose-dependent manner in normal horses. AAV8-eqIL-10 injected IVT in horses, at both LD (3.75×10^{11} vg) and HD (3.75×10^{12} vg), was well tolerated clinically, elicited few adverse events, and resulted in high concentrations of eqIL-10 in both the AH and VH of the eyes (see Figures 1, 2, and 3). However, transient aqueous flare (one LD horse) and conjunctival hyperemia (one LD and one HD horse), bilateral KP (both HD horses), peripapillary depigmentation (one HD horse), and mild (LD) to moderate (HD) histologic uveal LP cellular infiltration were observed, indicating that mild ocular inflammation developed following IVT AAV8-eqIL-10 dosing in normal horses (Figure 2).

The clinical and histopathologic ocular changes identified primarily in HD eyes after IVT dosing with AAV8-eqIL-10 may be driven by the elevated levels of IL-10, leading to a reduction in AAV clearance; by an immune response to the viral capsid or transgenic genome elements; or a combination of both.[36–38] Evaluation of an additional experimental group of horses IVT dosed with an equivalent AAV8 vector with a null mutation in the eqIL-10 open reading frame (i.e., similar packaged genome and size yet does not produce eqIL-10) would help determine whether the mild inflammation observed in the LD and HD eyes of this study was vector or transgene related, and such studies are under consideration. However, wild-type eqIL-10 was produced by the vector construct used in this study, which should not be seen as antigenic by the equine host. Interestingly, the only horses (LD 2 and HD 2) that developed clinical inflammatory abnormalities (i.e., conjunctival hyperemia or aqueous flare) were the ones that also had the highest IL-10 levels in the AH and VH (see Figure 3). Peak transduction occurs between 4 and 8 weeks after IVT injection,[39] which correlates to the time that inflammation was seen in these two horses. Although IL-10 has been demonstrated to have a broad anti-inflammatory effect,[23,27] it also may upregulate inflammation and promote mast cell development and degranulation, enhance cytotoxic T cell development, and be immunostimulatory to B lymphocytes.[40] Furthermore, IL-10 has been shown to help establish and perpetuate viral persistence by reducing the production of antiviral antibodies by B cells and reducing T cell proliferation, decreasing antigen presentation by dendritic cells, and reducing cytokine production.[40,41] Although all of these mechanisms play a role in decreasing inflammation and immune response, they also promote viral immune escape. IL-10 may also improve transgene expression by promoting viral persistence,[41] and in the case of the horses in this study, the increased IL-10 concentrations could have allowed improved transgene expression, leading to a self-perpetuating process that results in the high IL-10 levels observed in the AH and VH in this study. These findings suggest that the mild inflammation observed in this study may not be a vector-related immune response but may result from very high expression and levels of IL-10 in the eyes. While this mild inflammation is not likely significant enough to limit the efficacy of IL-10 in the treatment of uveitis, the high concentration of IL-10 found in the AH and VH and the presence of clinical inflammation supports the need for evaluation of lower doses of IVT AAV-eqIL-10 for the treatment of recurrent uveitis in horses.

AAV vectors have been used previously in experimental eqIL-10 gene therapy to treat orthopedic diseases.[35,42] One study involved the

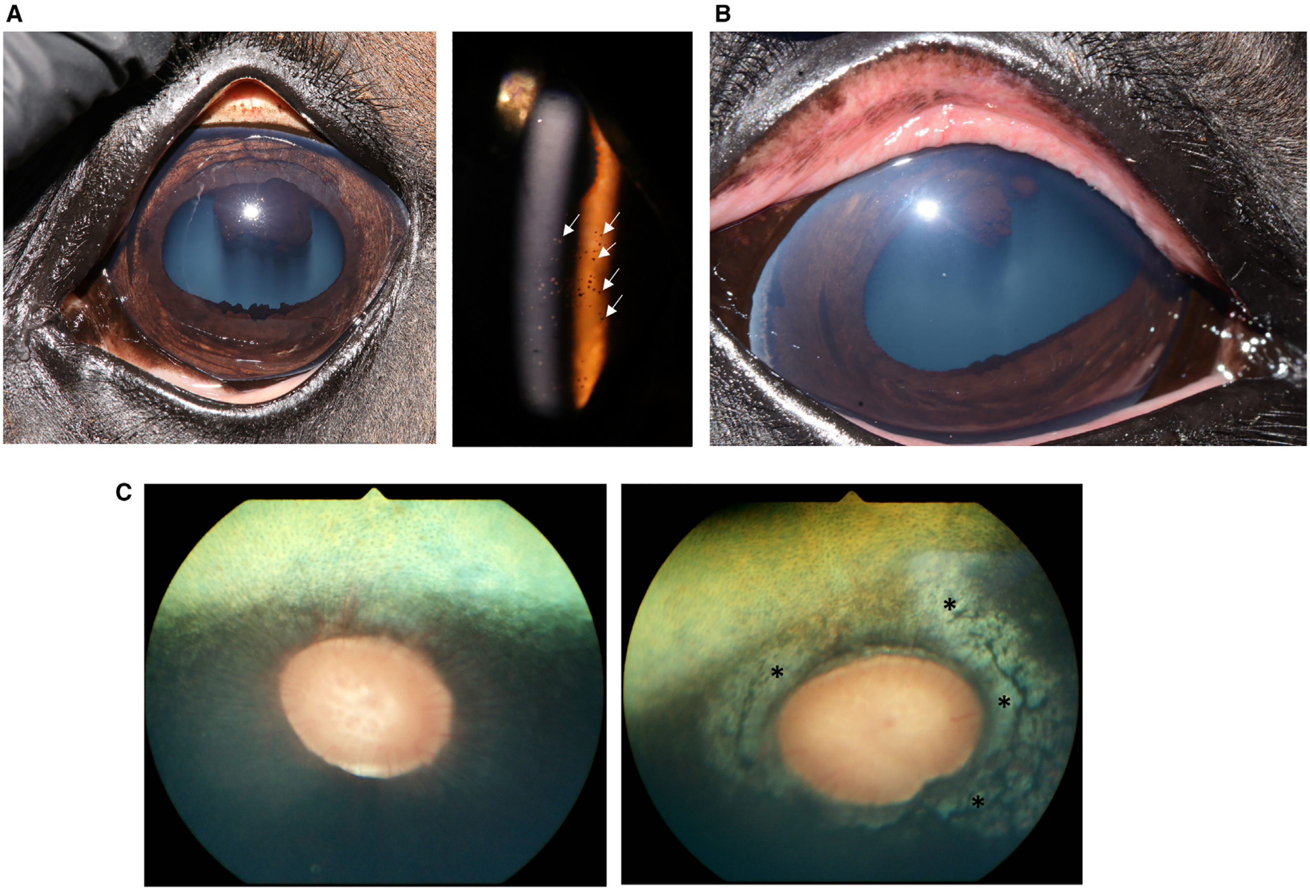

Figure 2. Adverse events

(A) Keratic precipitates (KP) were observed in both HD horses starting at 70 and 77 days after injection. On the left is a color image with faint diffuse pigmented areas in the inferior endothelial surface of the cornea. On the right, a slit lamp image demonstrates the pigmented KP on the endothelial corneal surface (white arrows). (B) In one low-dose horse (LD 2), conjunctival hyperemia and aqueous flare developed in one eye at days 28–49, which spontaneously resolved. (C) In one HD horse (HD 2), one eye developed peripapillary depigmentation (right image, *), initially observed on day 21 after injection, compared to a normal equine ocular fundus on the left.

intra-articular injection of 1×10^{12} vg of AAV5-IL-10 in normal equine carpal joints.[35] Similar to our ocular study, dosing with intra-articular AAV5-IL-10 showed sustained transgene expression in the joints and no significant differences in the clinical or histopathologic scores between saline or AAV5-IL-10 groups for 84 days post-injection. Furthermore, although vector shedding in urine and feces was not evaluated in that study, they did find that AAV5 vgs were present in the plasma of all AAV5 horses until day 4, with two horses having vgs detected at day 14, but an increase in plasma IL-10 was not detected.[35]

Normal physiologic levels of IL-10 in the equine eye were measured to be less than 9.06 pg/mL in the AH.[43] In horses with uveitis, this value increased 10-fold to a median of 114 pg/mL (<9.06–314.4 pg/mL).[43] Vitreous levels of IL-10 have not been previously reported in horses, but the VH concentrations were similar to aqueous levels in the vehicle-dosed horse in this study. IL-10 concentrations as low as 200 pg/mL have been shown to suppress CD4 T cells.[44] Samples from mouse eyes injected subretinally with AAV-IL-10 had IL-10 concentrations of 204–406 pg/mL (mean 308 pg/mL), sufficient to inhibit the development of experimental uveitis.[26] Therefore, if the targeted therapeutic level of IL-10 is 200–300 pg/mL (0.2–0.3 ng/mL), as suggested by the previous studies,[44,45] the IL-10 concentrations achieved in the horses of this study (LD and HD vitreous mean concentrations of 6.8 and 6.9 ng/mL, respectively) represent a 23- to 34-fold higher concentration of IL-10 than is needed to suppress inflammation in the eye. These data, paired with the effective doses observed in other local immunomodulatory studies,[46] support the importance of using lower doses of IVT AAV8-eqIL-10 when considering its use for treating clinical patients with uveitis. Of note, in this study, the BSS-injected horse had levels of IL-10 of 0.1 to 0.2 ng/mL, higher than previously reported in normal horses.[43] However, this higher concentration of IL-10 is hypothesized to be the result of inflammation from the IVT injection of BSS in the control eyes or possibly because of the differences in IL-10 testing methods with the previous study.[43] Importantly, significantly higher

Table 2. Vector shedding

Horse	Tissue[a,b]	eqIL-10 vgs detection[c]	Horse	Tissue[a,b]	eqIL-10 vgs detection[c]
Vehicle	tears D0 OS	–	LD 2	tears D0 OS	–
	tears D0 OD	–		tears D0 OD	–
	tears D1 OS	–		tears D1 OS	+
	tears D1 OD	–		tears D1 OD	+
	tears D3 OS	–		tears D3 OS	+
	tears D3 OD	–		tears D3 OD	–
	tears D14 OS	–		tears D14 OS	–
	tears D14 OD	–		tears D14 OD	–
LD 1	tears D0 OS	–	HD 1	tears D0 OS	–
	tears D0 OD	–		tears D0 OD	–
	tears D1 OS	+		tears D1 OS	–
	tears D1 OD	+		tears D1 OD	+
	tears D3 OS	–		tears D3 OS	–
	tears D3 OD	–		tears D3 OD	–
	tears D14 OS	–		tears D14 OS	–
	tears D14 OD	–		tears D14 OD	–
HD 2	tears D0 OS	–	HD 2	tears D3 OS	–
	tears D0 OD	–		tears D3 OD	–
	tears D1 OS	–		tears D14 OS	–
	tears D1 OD	–		tears D14 OD	–
All	urine D1	–	all	feces D1	–
	urine D3	–		feces D3	–
	urine D7	–		feces D7	–

[a]eqIL-10 shedding was measured from collected urine, feces, and tears samples.
[b]Tears from each eye on days (D) 0, 1, 3, and 14 were analyzed via PCR or qPCR. Urine and feces samples from days 1, 3, and 7 were analyzed via qPCR.
[c]+ indicates detection by PCR/qPCR; – indicates no detection by qPCR.

levels of equine IL-10 were measured in the eyes of horses receiving AAV-eqIL-10 relative to eyes receiving IVT BSS (Figure 3).

KP are clusters of inflammatory cells and proteinaceous debris on the endothelial surface of the cornea that are frequently observed in chronic uveitis.[5,6] A unique clinical finding in the HD treatment group of horses in this study was that there were minimal ophthalmic findings of inflammation before the development of peripapillary depigmentation (day 21), KPs (see Figure 2) first observed on day 71 after injection, and the moderate uveal cellular infiltrates observed on histology (day 86) (see Figure 4A). This clinical progression of subclinical ocular inflammation resulting in KPs and chorioretinopathy has been observed in experimental equine herpesvirus infections.[47] The similarities in ocular tissue response to viral infections in these two studies could implicate the viral vector, or its persistence from elevated levels of IL-10, as factors for the subclinical inflammation observed in this study.[35] Previous studies in murine models have identified that high-titer AAV can cause barrier dysfunction at the blood-brain barrier, allowing T cell infiltration.[48] Like the brain, the eye is often considered "immune privileged." IVT delivery of AAV vectors has previously been associated with increased immune response compared to other delivery routes.[49] The lymphoplasmacytic infiltration identified histopathologically could indicate that a similar barrier dysfunction occurred. This should be monitored closely in future evaluations in diseased animals, as barrier dysfunction and cellular infiltration are key mechanisms of uveitis. A dose-dependent inflammatory response has been reported previously in the primate eye following IVT injection of an AAV vector, both locally and with a systemic humoral response.[50–52] While a secondary analysis of a phase 1/2 clinical trial of a therapy utilizing AAV2-ND4 found no connection between dose or humoral response with local inflammation,[53] a different phase 1/2a clinical trial evaluating AAV8-*RS1* in humans exhibited further support for a dose-dependent inflammatory response.[54] In the latter, the reactions were well controlled with topical and systemic anti-inflammatory medications. While both dose groups of horses in this study had changes in ocular examinations from baseline, findings in the LD group were mild, self-limiting, and considered non-adverse.

IVT injection of AAV8-eqIL-10 resulted in a wide distribution of vgs in the equine eye, including targeted ocular tissues such as the retina, ICB, conjunctiva, cornea, choroid, AH, and VH in both treatment groups (see Figure 5). This study demonstrated successful

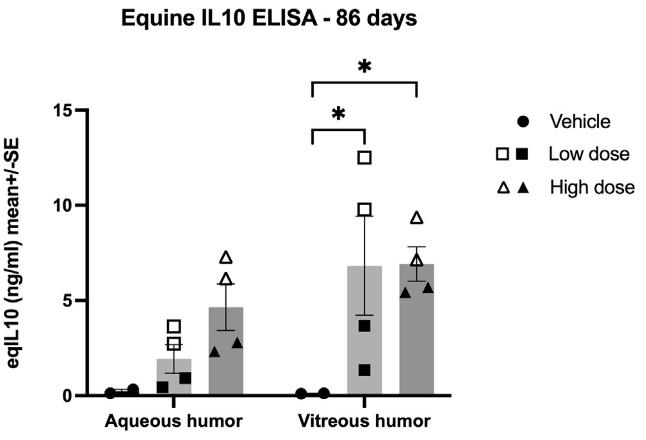

Figure 3. eqIL-10 ELISA

eqIL-10 was measured in the aqueous (AH) and vitreous humor (VH) at 86 days after injection of AAV-eqIL-10 IVT. IL-10 concentrations in the vitreous were significantly elevated in both the LD (3.75e−11 vg, ■□) and high dose (3.75e−12 vg, ▲Δ) groups compared to the saline vehicle group (*$p < 0.05$; two-way ANOVA with Tukey's). Data represented as mean ± SEM.

transduction of ocular tissue, with minimal off-target effects following IVT injection, which is consistent with the previously reported data from our laboratories in the rodent model showing that AAV gene transfer can successfully induce immunomodulation with limited systemic effect, even when using equine IL-10 in rodents.[28,29] Concordantly, systemic distribution was not identified in any sampled organs except for the liver, where vgs were detected in one horse from the LD group (LD 2) and both horses from the HD group, and the kidney (a single HD horse) (see Table 3). This is consistent with previous studies that have detected hepatic AAV8 after IVT therapy in nonhuman primates.[55] Despite the difference in dose, there was no significant difference in vgs detected in the cornea and retina. These discrepancies are likely due to the portion of the tissue sampled or a variation in individual horse immune response. When evaluating new therapy in herd-based species such as horses, the environmental exposure of a recombinant virus to other individuals in the herd and or handlers is an important consideration. The limited time of shedding of vgs in tears after IVT injection of AAV8-eqIL-10 in this study and the lack of vgs detected in feces and urine reduce herd and environmental exposure concerns.

While ERU is an important equine health concern, its high prevalence (2%–5%) and similarities to human NIU make it an important large animal model for innovative therapeutics.[6] While other inducible models are available, the only well-described spontaneous model of NIU is ERU. Similarities between the human and equine diseases include relapsing/remitting clinical presentation, similar autoantigens, and their immunopathology.[7] Both diseases (ERU and NIU) involve the breakdown of the blood-ocular barrier and result in T cell-mediated tissue damage. The longer lifespan of horses compared to traditional induced rodent models also allows for long-term follow-up, which is another future direction of the research of this therapeutic. Furthermore, these preliminary studies reported herein are particularly important in translating results from the EAU rodent efficacy studies to larger animals.

The limitations to this study include the low number of horses evaluated and the sex predilection toward female horses, which was unavoidable due to the available donated population. While there was evidence of inflammation in both the LD and the HD groups, the LD horses appeared clinically normal at the end of the study. The successful transduction of ocular tissue and high production of eqIL-10 indicates that the dose may be lowered, possibly 20- to 30-fold, and still deliver effective levels of IL-10, which could decrease or eliminate any observed adverse effects. Future directions include assessing lower dosages of the vgs, increasing study numbers and duration, and evaluating the therapeutic in horses with clinical uveitis. While the treatment groups did not have a statistically significant difference in IL-10, one horse in the LD group obtained IL-10 levels like those of the HD group (see Figure 3). This horse was also the only other horse with flare seen transiently, elevating the ocular score (see Figure 1A), which could implicate the IL-10 level rather than the dose of vgs. However, the similarity of clinical signs with those seen in herpesvirus infections, the history of inflammatory stimulation with AAV8 administered IVT in other species, and the lack of significant differences in IL-10 levels between dose groups despite the difference in inflammation leads to the suggestion of future evaluation of a null viral vector (as previously described, producing no IL-10) and IL-10 protein control. It is hoped that this would elucidate the pathophysiology of the inflammation seen in these horses (vector-mediated versus IL-10-mediated immune response).

In conclusion, this first-of-its-kind evaluation of AAV ocular gene therapy in horses has shown effective production of IL-10 following IVT injection of AAV8-eqIL-10 in healthy horses. While mild to moderate local inflammatory changes were observed with dose-dependent effects, no systemic concerns were identified throughout 86 days after dosing. Further investigation of lower doses of IVT AAV8-eqIL-10 therapy is an important next step toward a safe and effective one-time treatment of equine uveitis, with broader implications for the treatment of NIU in humans.

MATERIALS AND METHODS

AAV8-eqIL-10 design and manufacture

Equine IL-10 cDNA was codon optimized (GenScript USA, Piscataway, NJ) and cloned downstream of the cytomegalovirus promoter in a self-complementary AAV production plasmid. AAV8-eqIL-10 was produced by The University of North Carolina Vector Core and characterized, as previously described.[29] AAV8 vectors were purified by iodixanol gradient centrifugation followed by Q HP ion-exchange chromatography (Cytiva, Marlborough, MA). Titration and quantification were performed using an inverted terminal repeat sequence (ITR) qPCR assay (forward ITR primer, 5′-GGAACCCCT AGTGATGGAGTT; reverse ITR primer, 5′-CGGCCTCAGTGAG CGA).[56]

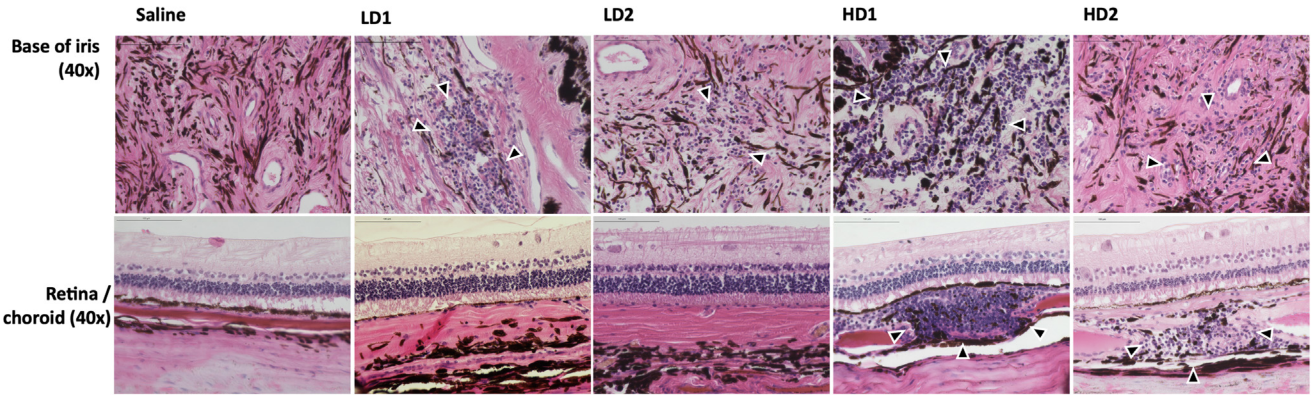

Figure 4. Representative histopathology

Saline-injected eyes had no inflammatory changes. LD eyes (LD1 and LD2) were injected with 3.75e−11 vgs and had slight, scattered lymphocytic/plasmocytic (LP) cells in the ciliary body, small foci of lymphocytes in the posterior iris (arrowheads), and normal retina in both eyes. In the HD eyes (HD1 and HD2), injected with 3.75e−12 vgs, there was diffuse moderate LP infiltration into the iris (arrowheads), ciliary body processes, and pars plana. Also, multifocal, diffuse inferior, moderate-sized nests of LP infiltrated the choroid (arrowheads), but the overlying retina appeared normal.

Horses, dosing, clinical examination, and in-life sample collection

The animals used in this study adhered to the Association for Research in Vision and Ophthalmology statement for the use of animals in ophthalmic and vision research. The care and use of animals in this study were also approved and monitored by the North Carolina State University Institutional Animal Care and Use (approval no. 22-349). Horses used were donated to North Carolina State University and underwent physical and ocular examinations, complete blood counts, and serum chemistry analysis, all of which were either normal or had abnormalities that were deemed not to influence the study (see Table S1). Horses were maintained on pasture in the North Carolina State Laboratory Animal Resources facilities. Five horses were randomly divided into three groups. After 7 days of acclimation, horses received IVT injections as described in the next section. OEs were performed on days 0 (before injection), 1, 3, and 7 and weekly until day 86 post-IVT injection. Tears were collected in both eyes of each horse by saturating a Schirmer tear test strip (Alcon Laboratories, Fort Worth, TX) with tears, sealing the strip in a labeled Eppendorf tube, and storing at −80°C. Urine, fresh stool, serum, and whole-blood samples were collected and stored at −80°C until processing. The collection of samples was performed on days 0, 1, 3, and 7, and then weekly through day 86 (Figure S1).

IVT injections

Regional anesthesia (i.e., frontal and auriculopalpebral nerve blocks) was performed to facilitate analgesia and akinesia of the ocular globe and eyelids (3 mL of 2% lidocaine HCl) and topical 0.5% proparacaine HCl. Dilute povidone-iodine solution (5%), followed by irrigation with sterile saline, was used to prepare the ocular surface before IVT injection. Injection of either control (BSS, Alcon Laboratories), LD (3.75×10^{11} vg) ($n = 2$ eyes), or HD (3.75×10^{12} vg) in a volume of 500 μL was performed using a 27G needle (Sterican, B. Braun, Bethlehem, PA) and a 1-mL syringe. The needle was inserted 8–10 mm posterior to the superior-temporal limbus at the pars plana and directed toward the optic disc to avoid contact with the lens, and the vector was delivered to the central vitreous. After injection, a topical application (100 μL) of bacitracin-neomycin-gramicidin ophthalmic solution was applied to the surface of the eye.

OEs

Results of complete ophthalmic examinations (slit lamp biomicroscopy, indirect ophthalmoscopy, IOP, clinical photographs) were recorded to evaluate inflammation in the eye. Examinations were performed by the primary investigator, who is board certified in veterinary ophthalmology and ocular toxicology. Inflammatory scores were recorded using a scoring system, which is described for the use of assessment of ocular inflammation in animals during toxicological examinations using a semiquantitative numerical scale.[57] Cumulative scores were calculated to provide an overall score per eye per examination day. IOP was measured in both eyes at the OE time points. IOP was measured using a rebound tonometer (iCare Tonometer, Espoo, Finland) without the use of a topical anesthetic. The tip of the tonometer probe was directed to gently contact the central cornea. The average IOP shown on the display was recorded. This procedure was then repeated two additional times, and the measurements were recorded and averaged.

Euthanasia and sample collection

Eighty-six days after IVT injections, the horses were euthanized by intravenous barbiturate injection. At euthanasia, the left eye of each animal was removed, fixed in Davidson's solution at 4°C for 48 h, transferred to 70% ethanol, and processed for H&E histology or immunostained to detect CD3. For immunostaining, antigen retrieval was performed using the pressure cooker method (Citra pH = 6, Biogenex, Fremont, CA) followed by an endogenous peroxidase block performed for 20 min at room temperature, followed by

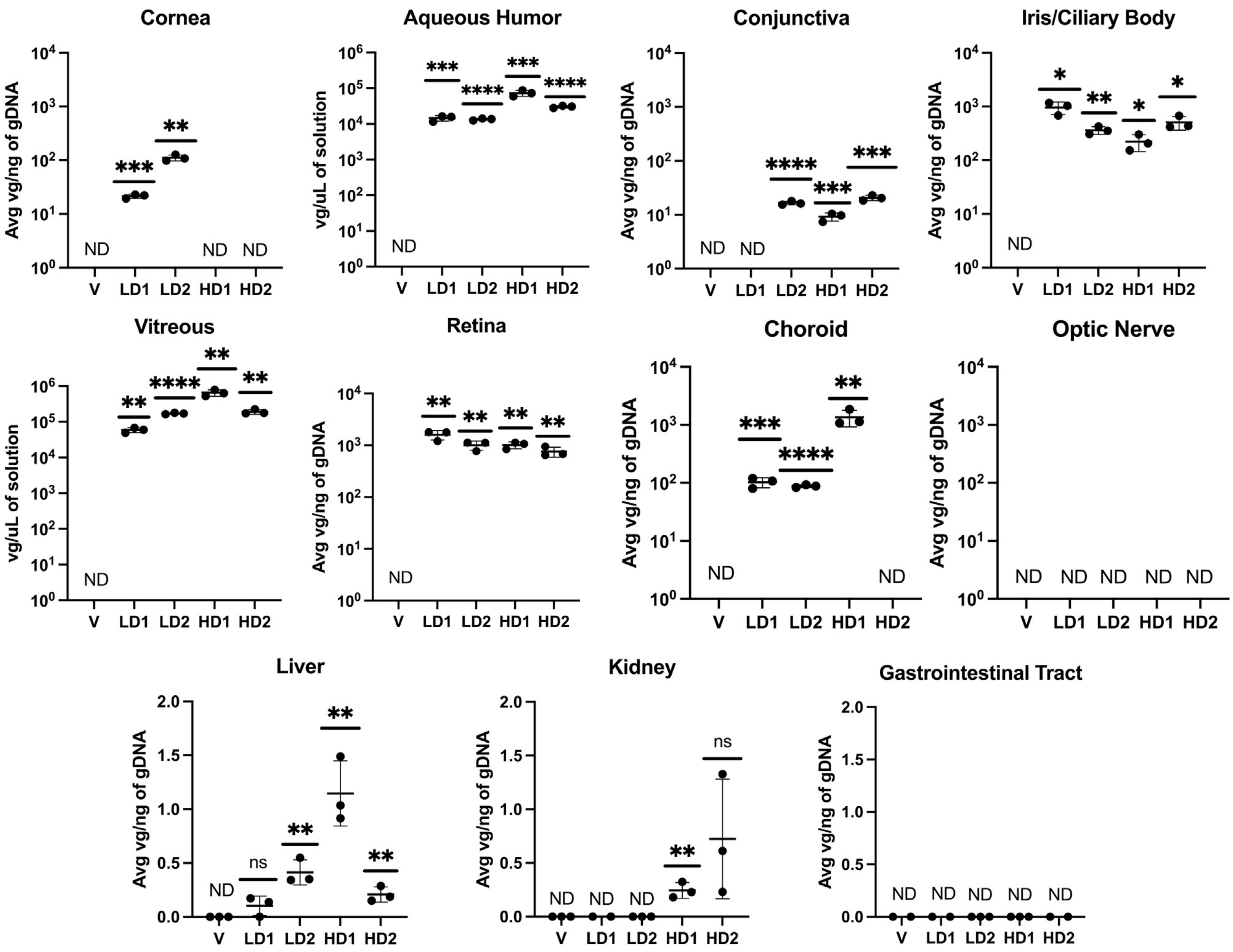

Figure 5. vg distribution of eqIL-10

The ocular and systemic distribution of AAV8-eqIL-10 vgs were quantified via qPCR on tissue recovered following euthanasia from all horses. Each point represents one technical replicate ($n = 3$), with mean ± standard deviation noted. Findings are portrayed as the average vg/ng of recovered genomic DNA. HD, high-dose treated; LD, low-dose treated; V, vehicle treated. Significance levels were determined between that of the treated and untreated samples using an unpaired t test (ns, $p > 0.05$; *$p < 0.05$; **$p < 0.01$; ***$p < 0.001$; ****$p < 0.0001$). For the AH and VH, findings are portrayed as the average vg/μL of recovered solution. Significance levels were determined between that of the treated and untreated samples using an unpaired t test (ns, $p > 0.05$; *$p < 0.05$; **$p < 0.01$; ***$p < 0.001$; ****$p < 0.0001$).

Fc blocker (Innovex Biosciences, Richmond, CA) for 30 min. The primary antibody used was a rabbit polyclonal (30 min, Agilent Dako A0452, Santa Clara, CA) and the secondary antibody was a polyclonal rabbit (45 min, VectorLabs HRP, Newark, CA). 3,3’-Diaminobenzidine chromogen was then applied (3 min, Vector Labs), followed by a hematoxylin counterstain (5 min). The resulting slides were examined by light microscopy. The right eye from each horse was removed and dissected to isolate the conjunctiva, cornea, ICB, vitreous, retina, choroid, optic nerve, AH, and extraocular muscles in which DNA was recovered for vector biodistribution using qPCR. Additionally, samples of whole blood, brain, submandibular lymph nodes, nasal mucosa, heart, liver, spleen, kidney, and skeletal muscle were collected for vector biodistribution studies using qPCR and endpoint PCR.

eqIL-10 ELISA

The abundance of eqIL-10 was measured in the AH and VH by equine-specific ELISA (Equine IL-10 ELISA ab155466, Abcam, Waltham, MA). VH was centrifuged as previously described,[42] and analysis was performed on the supernatant. AH and VH were analyzed according to manufacturer protocol in duplicate. Absorbance was detected using a microplate reader (Synergy 2, BioTek, Portland, ME). A standard curve was generated using serial dilutions of kit provided recombinant eqIL-10 and analyzed using GraphPad Prism 10 (Boston, MA).

Ocular distribution

DNA from portions of the AH, VH, choroid, cornea, conjunctiva, optic nerve, retina, and ICB were isolated with a DNeasy Blood and Tissue Kit following the manufacturer’s instructions (Qiagen, Valencia,

Table 3. Peripheral organ distribution of eqIL-10 genomes

Tissue[a]	eqIL-10 detection[b]
Heart	–
Spleen	–
Brain	–
Nasal mucosa	–
Skeletal muscle	–
Submandibular lymph node	–
Whole blood (days 3 and 7)	–
Liver	+/LD 2, HD 1, HD 2
Kidney	+/HD 1
Gastrointestinal tract	–

[a]Peripheral organ distribution was measured from collected tissue after euthanasia via qPCR and endpoint PCR.
[b]– indicates no detection by PCR/qPCR; +/X indicates detection by PCR/qPCR in subject X.

CA). The ocular distribution of AAV8-eqIL-10 vgs was determined through qPCR reactions utilizing a TaqMan Universal PCR Mastermix (Thermo Fisher Scientific, Waltham, MA) and a custom 20× TaqMan primer/probe set specific to eqIL-10 DNA (forward: 5′-CTGTGCTAC CTGGTGTTCCT-3′; reverse: 5′-TACTTCCTGGTCGACCTGTT-3′; probe: 5′-CCGCGGCACCCAGAGCGAGA-3′). A standard curve was generated using eight 1:10 serial dilutions with a known concentration of a plasmid encoding eqIL-10. TaqMan reactions were carried out in 20-μL volumes, including 1 μL of ocular sample, in a 96-well format and quantified in triplicate for each sample. Thermal cycling was completed on the StepOnePlus Real-Time PCR System (Applied Biosystems, Waltham, MA) with the following parameters: 50°C for 2 min and 95°C for 10 min, followed by 45 cycles of 95°C for 1 min, 58°C for 30 s, and 72°C for 1 s. The vgs were quantified as previously described,[28] and biodistribution data are shown as AAV8-eqIL-10 vg/ng DNA and vg/μL solution for AH/VH samples. Samples quantified below the limit of detection are shown as not detected (ND).

Peripheral organ distribution

The peripheral organ distribution of eqIL-10 genomes was analyzed by isolating DNA from samples collected and performing endpoint or qPCR. Total DNA from each sample was isolated with the DNeasy Blood and Tissue Kit (catalog no. 69504, Qiagen). Briefly, tissues were lysed overnight at 56°C, and fluids were incubated at 56°C for 10 min. Samples were bound to a DNA spin column, washed, and eluted. For heart, spleen, brain, nasal mucosa, submandibular lymph node, skeletal muscle, and whole-blood samples (days 3 and 7), endpoint PCR was performed using 1 μL isolated DNA as a template with an annealing temperature of 59°C, 35 cycles, 19-s extension time, and the following primers: forward 5′-TCACATGCTGCACGAGCTGA-3′ and reverse 5′-ACGGCCTTGCTCTTGTTCTC-3′. Amplicons were run on a 2% agarose gel, and the presence of a 303-bp-size band indicated a positive result in that tissue. This assay's limit of detection was determined to be 0.3 pg using a plasmid encoding eqIL-10. Liver, kidney, and GI tract distribution of AAV8-eqIL-10 genomes were quantified through qPCR in the same manner as described for ocular distribution.

Viral shedding: Tears, urine, feces

Viral shedding of eqIL-10 genomes was investigated through PCR/qPCR analysis of tears, urine, and feces samples from all horses. Samples were collected before and after injection of AAV8-eqIL-10. DNA from each sample was isolated with the DNeasy Blood and Tissue kit (catalog no. 69504, Qiagen) per kit instructions. The distribution of eqIL-10 genomes in urine samples was quantified by PCR as described for peripheral organ distribution. The distribution of eqIL-10 genomes in feces and tears samples was quantified by qPCR as described for ocular distribution. Samples were run in triplicate.

Neutralizing antibody assay

The neutralizing antibody titers were evaluated as previously described with modifications.[58] Briefly, HEK293 cells were seeded into 48-well plates at 100,000 cells/well in duplicate 24 h before vector transduction. Serum dilutions (1:1 and serially diluted from 1:2 to 1:128 in Dulbecco's PBS, 1 μL serum dilutions/well) were incubated with the same volume of AAV8-chicken β-actin-luciferase (1×10^8 vg/well, prepared at The University of North Carolina Vector Core) for 1 h at 4°C. The mixture was added to cells and incubated for 48 h at 37°C; then, cell lysates were harvested with passive lysis buffer (E194A, Promega, Fitchburg, WI) and tested for luciferase activity using luciferase assay reagent (E1483, Promega). Neutralizing antibody titers were defined as the highest dilution that demonstrated decreased luciferase activity that was less than or equal to 50% compared to serum-free controls.

Statistical analysis

Animal numbers were estimated using power statistics and a clinical inflammatory scoring system from previous studies of the use of AAV-eqIL-10 in EAU.[28,29] With target endpoints of less than 4 cumulative ocular inflammatory score differences between groups and an estimated SD of 1.8, using a $p < 0.05$, $n = 4$ eyes/group will provide a power of 0.83. Wilcoxon tests (nonparametric) were used to determine significance among groups. A mixed-effects model was used for the analysis of inter-eye correlation. Pairwise Wilcoxon tests were used to compare clinical scores, and an ANOVA with Tukey's post hoc analysis was used to compare IOP and biodistribution data among groups. Computerized statistical software JMP Pro version 16.0 and GraphPad Prism 10 were used for calculations.

DATA AND CODE AVAILABILITY

The data that support the findings of this study are available from the corresponding author upon request.

ACKNOWLEDGMENTS

This work was supported by the North Carolina Biotechnology Center through a Translational Research Grant. Production support was provided by the UNC Vector Core for production of the vector. Stipend support for K.Y. was provided by NIH T32OD011130. This project was also supported by Erin Barr, who served as the project manager, and

Bryan Stallings, who provided technical assistance. Further support was provided by Jori Vasgaard and the staff at the North Carolina State University Reedy Creek Equine Farm for the maintenance of daily attentive care of the animals involved in the project.

AUTHOR CONTRIBUTIONS

Conceptualization, B.C.G. and M.L.H. Data acquisition, K.Y., N.H., B.C.G., and J.H.S. Data analysis and interpretation, B.C.G., M.L.H., K.Y., T.H., N.V., T.F.M., and N.H. Manuscript preparation, K.Y., B.C.G., M.L.H., T.F.M., and N.V. Manuscript editing and review, B.C.G., M.L.H., K.Y., T.H., N.V., and T.F.M.

DECLARATION OF INTERESTS

B.C.G. and M.L.H. hold a provisional patent for the clinical therapeutic investigated.

SUPPLEMENTAL INFORMATION

Supplemental information can be found online at https://doi.org/10.1016/j.omtm.2024.101360.

REFERENCES

1. Jones, N.P. (2015). The Manchester Uveitis Clinic: The First 3000 Patients-Epidemiology and Casemix: Ocular Immunology & Inflammation. Ocul. Immunol. Inflamm. *23*, 118–126. https://doi.org/10.3109/09273948.2013.855799.
2. Thorne, J.E., Suhler, E., Skup, M., Tari, S., Macaulay, D., Chao, J., and Ganguli, A. (2016). Prevalence of Noninfectious Uveitis in the United States: A Claims-Based Analysis. JAMA Ophthalmol. *134*, 1237–1245. https://doi.org/10.1001/jamaophthalmol.2016.3229.
3. Abdulaal, M.R., Abiad, B.H., and Hamam, R.N. (2015). Uveitis in the Aging Eye: Incidence, Patterns, and Differential Diagnosis. J. Ophthalmol. *2015*, 509456. https://doi.org/10.1155/2015/509456.
4. Gritz, D.C., and Wong, I.G. (2004). Incidence and prevalence of uveitis in Northern California: The Northern California Epidemiology of Uveitis Study. Ophthalmology *111*, 491–500. https://doi.org/10.1016/j.ophtha.2003.06.014.
5. Gerding, J.C., and Gilger, B.C. (2016). Prognosis and impact of equine recurrent uveitis. Equine Vet. J. *48*, 290–298. https://doi.org/10.1111/evj.12451.
6. Deeg, C.A., Hauck, S.M., Amann, B., Pompetzki, D., Altmann, F., Raith, A., Schmalzl, T., Stangassinger, M., and Ueffing, M. (2008). Equine Recurrent Uveitis – A Spontaneous Horse Model of Uveitis. Ophthalmic Res. *40*, 151–153. https://doi.org/10.1159/000119867.
7. Witkowski, L., Cywinska, A., Paschalis-Trela, K., Crisman, M., and Kita, J. (2016). Multiple etiologies of equine recurrent uveitis – A natural model for human autoimmune uveitis: A brief review. Comp. Immunol. Microbiol. Infect. Dis. *44*, 14–20. https://doi.org/10.1016/j.cimid.2015.11.004.
8. Malalana, F., Stylianides, A., and McGowan, C. (2015). Equine recurrent uveitis: Human and equine perspectives. Vet. J. *206*, 22–29. https://doi.org/10.1016/j.tvjl.2015.06.017.
9. Willermain, F., Rosenbaum, J.T., Bodaghi, B., Rosenzweig, H.L., Childers, S., Behrend, T., Wildner, G., and Dick, A.D. (2012). Interplay between innate and adaptive immunity in the development of non-infectious uveitis. Prog. Retin. Eye Res. *31*, 182–194. https://doi.org/10.1016/j.preteyeres.2011.11.004.
10. Srivastava, A., Rajappa, M., and Kaur, J. (2010). Uveitis: Mechanisms and recent advances in therapy. Clin. Chim. Acta *411*, 1165–1171. https://doi.org/10.1016/j.cca.2010.04.017.
11. Jones, N.P. (2015). The Manchester Uveitis Clinic: the first 3000 patients, 2: uveitis manifestations, complications, medical and surgical management. Ocul. Immunol. Inflamm. *23*, 127–134.
12. Bastola, P., Song, L., Gilger, B.C., and Hirsch, M.L. (2020). Adeno-Associated Virus Mediated Gene Therapy for Corneal Diseases. Pharmaceutics *12*, 767. https://doi.org/10.3390/pharmaceutics12080767.
13. Miraldi Utz, V., Coussa, R.G., Antaki, F., and Traboulsi, E.I. (2018). Gene therapy for RPE65-related retinal disease: Ophthalmic Genetics. Ophthalmic Genet. *39*, 671–677. https://doi.org/10.1080/13816810.2018.1533027.
14. Patel, U., Boucher, M., de Léséleuc, L., and Visintini, S. (2016). Voretigene Neparvovec: An Emerging Gene Therapy for the Treatment of Inherited Blindness. In CADTH Issues in Emerging Health Technologies CADTH Horizon Scans (Canadian Agency for Drugs and Technologies in Health).
15. Li, C., and Samulski, R.J. (2020). Engineering adeno-associated virus vectors for gene therapy. Nat. Rev. Genet. *21*, 255–272. https://doi.org/10.1038/s41576-019-0205-4.
16. Tsai, M.-L., Horng, C.-T., Chen, S.-L., Xiao, X., Wang, C.-H., and Tsao, Y.-P. (2009). Suppression of experimental uveitis by a recombinant adeno-associated virus vector encoding interleukin-1 receptor antagonist. Mol. Vis. *15*, 1542–1552.
17. Chan, Y.K., Dick, A.D., Hall, S.M., Langmann, T., Scribner, C.L., and Mansfield, B.C.; Ocular Gene Therapy Inflammation Working Group (2021). Inflammation in Viral Vector-Mediated Ocular Gene Therapy: A Review and Report From a Workshop Hosted by the Foundation Fighting Blindness, 9/2020. Transl. Vis. Sci. Technol. *10*, 3. https://doi.org/10.1167/tvst.10.4.3.
18. Bogner, B., Boye, S.L., Min, S.H., Peterson, J.J., Ruan, Q., Zhang, Z., Reitsamer, H.A., Hauswirth, W.W., and Boye, S.E. (2015). Capsid Mutated Adeno-Associated Virus Delivered to the Anterior Chamber Results in Efficient Transduction of Trabecular Meshwork in Mouse and Rat. PLoS One *10*, e0128759. https://doi.org/10.1371/journal.pone.0128759.
19. Song, L., Llanga, T., Conatser, L.M., Zaric, V., Gilger, B.C., and Hirsch, M.L. (2018). Serotype survey of AAV gene delivery via subconjunctival injection in mice. Gene Ther. *25*, 402–414. https://doi.org/10.1038/s41434-018-0035-6.
20. Buie, L.K., Rasmussen, C.A., Porterfield, E.C., Ramgolam, V.S., Choi, V.W., Markovic-Plese, S., Samulski, R.J., Kaufman, P.L., and Borrás, T. (2010). Self-complementary AAV Virus (scAAV) Safe and Long-term Gene Transfer in the Trabecular Meshwork of Living Rats and Monkeys. Invest. Ophthalmol. Vis. Sci. *51*, 236–248. https://doi.org/10.1167/iovs.09-3847.
21. Moore, K.W., de Waal Malefyt, R., Coffman, R.L., and O'Garra, A. (2001). Interleukin-10 and the Interleukin-10 Receptor. Annu. Rev. Immunol. *19*, 683–765. https://doi.org/10.1146/annurev.immunol.19.1.683.
22. Iyer, S.S., and Cheng, G. (2012). Role of Interleukin 10 Transcriptional Regulation in Inflammation and Autoimmune Disease. Crit. Rev. Immunol. *32*, 23–63.
23. Couper, K.N., Blount, D.G., and Riley, E.M. (2008). IL-10: The Master Regulator of Immunity to Infection. J. Immunol. *180*, 5771–5777. https://doi.org/10.4049/jimmunol.180.9.5771.
24. O'Garra, A., Barrat, F.J., Castro, A.G., Vicari, A., and Hawrylowicz, C. (2008). Strategies for use of IL-10 or its antagonists in human disease. Immunol. Rev. *223*, 114–131. https://doi.org/10.1111/j.1600-065X.2008.00635.x.
25. Trittibach, P., Barker, S.E., Broderick, C.A., Natkunarajah, M., Duran, Y., Robbie, S.J., Bainbridge, J.W.B., Smith, A.J., Sarra, G.-M., Dick, A.D., and Ali, R.R. (2008). Lentiviral-Vector Mediated Expression of Murine IL-1 Receptor Antagonist or IL-10 Reduces the Severity of Endotoxin-Induced Uveitis. Gene Ther. *15*, 1478–1488. https://doi.org/10.1038/gt.2008.109.
26. Broderick, C.A., Smith, A.J., Balaggan, K.S., Georgiadis, A., Buch, P.K., Trittibach, P.C., Barker, S.E., Sarra, G.-M., Thrasher, A.J., Dick, A.D., and Ali, R.R. (2005). Local administration of an adeno-associated viral vector expressing IL-10 reduces monocyte infiltration and subsequent photoreceptor damage during experimental autoimmune uveitis. Mol. Ther. *12*, 369–373.
27. Ghasemi, H., Ghazanfari, T., Yaraee, R., Owlia, P., Hassan, Z.M., and Faghihzadeh, S. (2012). Roles of IL-10 in ocular inflammations: a review. Ocul. Immunol. Inflamm. *20*, 406–418.
28. Crabtree, E., Uribe, K., Smith, S.M., Roberts, D., Salmon, J.H., Bower, J.J., Song, L., Bastola, P., Hirsch, M.L., and Gilger, B.C. (2022). Inhibition of experimental autoimmune uveitis by intravitreal AAV-Equine-IL10 gene therapy. PLoS One *17*, e0270972. https://doi.org/10.1371/journal.pone.0270972.
29. Crabtree, E., Song, L., Llanga, T., Bower, J.J., Cullen, M., Salmon, J.H., Hirsch, M.L., and Gilger, B.C. (2019). AAV-mediated expression of HLA-G1/5 reduces severity of experimental autoimmune uveitis. Sci. Rep. *9*, 19864. https://doi.org/10.1038/s41598-019-56462-3.
30. Patipa, L.A., Sherlock, C.E., Witte, S.H., Pirie, G.D., Berghaus, R.D., and Peroni, J.F. (2012). Risk factors for colic in equids hospitalized for ocular disease. J. Am. Vet. Med. Assoc. *240*, 1488–1493.
31. Scherrer, N.M., Lassaline, M., Richardson, D.W., and Stefanovski, D. (2016). Interval prevalence of and factors associated with colic in horses hospitalized for ocular or orthopedic disease. J. Am. Vet. Med. Assoc. *249*, 90–95.

32. Curto, E.M., Griffith, E.H., Posner, L.P., Walsh, K.T., Balko, J.A., and Gilger, B.C. (2018). Factors associated with postoperative complications in healthy horses after general anesthesia for ophthalmic versus non-ophthalmic procedures: 556 cases (2012–2014). J. Am. Vet. Med. Assoc. *252*, 1113–1119.

33. Beebe, A.M., Cua, D.J., and de Waal Malefyt, R. (2002). The role of interleukin-10 in autoimmune disease: systemic lupus erythematosus (SLE) and multiple sclerosis (MS). Cytokine Growth Factor Rev. *13*, 403–412. https://doi.org/10.1016/S1359-6101(02)00025-4.

34. Li, R., Zhang, L., Peng, C., Lu, Y., Liu, Z., Xu, X., Wang, C., Hu, R., Tan, W., Zhou, L., et al. (2024). Chronic Expression of Interleukin-10 Transgene Modulates Cardiac Sympathetic Ganglion Resulting in Reduced Ventricular Arrhythmia. Hum. Gene Ther. *35*, 114–122. https://doi.org/10.1089/hum.2023.160.

35. Moss, K.L., Jiang, Z., Dodson, M.E., Linardi, R.L., Haughan, J., Gale, A.L., Grzybowski, C., Engiles, J.E., Stefanovski, D., Robinson, M.A., and Ortved, K.F. (2020). Sustained Interleukin-10 Transgene Expression Following Intra-Articular AAV5-IL-10 Administration to Horses. Hum. Gene Ther. *31*, 110–118. https://doi.org/10.1089/hum.2019.195.

36. Li, C., and Samulski, R.J. (2020). Engineering adeno-associated virus vectors for gene therapy. Nat. Rev. Genet. *21*, 255–272.

37. Xiong, W., Wu, D.M., Xue, Y., Wang, S.K., Chung, M.J., Ji, X., Rana, P., Zhao, S.R., Mai, S., and Cepko, C.L. (2019). AAV cis-regulatory sequences are correlated with ocular toxicity. Proc. Natl. Acad. Sci. USA *116*, 5785–5794.

38. Mehta, N., Robbins, D.A., and Yiu, G. (2021). Ocular inflammation and treatment emergent adverse events in retinal gene therapy. Int. Ophthalmol. Clin. *61*, 151–177.

39. Katada, Y., Kobayashi, K., Tsubota, K., and Kurihara, T. (2019). Evaluation of AAV-DJ vector for retinal gene therapy. PeerJ *7*, e6317.

40. Conti, P., Kempuraj, D., Kandere, K., Di Gioacchino, M., Barbacane, R.C., Castellani, M.L., Felaco, M., Boucher, W., Letourneau, R., and Theoharides, T.C. (2003). IL-10, an inflammatory/inhibitory cytokine, but not always. Immunol. Lett. *86*, 123–129.

41. Wilson, E.B., and Brooks, D.G. (2011). The Role of IL-10 in Regulating Immunity to Persistent Viral Infections (Negative Co-Receptors and Ligands), pp. 39–65.

42. Abu El-Asrar, A.M., Mohammad, G., Nawaz, M.I., Siddiquei, M.M., Van Den Eynde, K., Mousa, A., De Hertogh, G., and Opdenakker, G. (2013). Relationship between Vitreous Levels of Matrix Metalloproteinases and Vascular Endothelial Growth Factor in Proliferative Diabetic Retinopathy. PLoS One *8*, e85857. https://doi.org/10.1371/journal.pone.0085857.

43. Curto, E., Messenger, K.M., Salmon, J.H., and Gilger, B.C. (2016). Cytokine and chemokine profiles of aqueous humor and serum in horses with uveitis measured using multiplex bead immunoassay analysis. Vet. Immunol. Immunopathol. *182*, 43–51. https://doi.org/10.1016/j.vetimm.2016.09.008.

44. Wang, Y., Yu, P., Li, Y., Zhao, Z., Wu, X., Zhang, L., Feng, J., and Hong, J.-S. (2021). Early-Released Interleukin-10 Significantly Inhibits Lipopolysaccharide-Elicited Neuroinflammation In Vitro. Cells *10*, 2173. https://doi.org/10.3390/cells10092173.

45. Fang, I.-M., Lin, C.-P., Yang, C.-H., Chiang, B.-L., Yang, C.-M., Chau, L.-Y., and Chen, M.-S. (2005). Inhibition of experimental autoimmune anterior uveitis by adenovirus-mediated transfer of the interleukin-10 gene. J. Ocul. Pharmacol. Therapeut. *21*, 420–428.

46. Huynh, T., Reed, C., Blackwell, Z., Phelps, P., Herrera, L.C.P., Almodovar, J., Zaharoff, D.A., and Wolchok, J. (2023). Local IL-10 delivery modulates the immune response and enhances repair of volumetric muscle loss muscle injury. Sci. Rep. *13*, 1983. https://doi.org/10.1038/s41598-023-27981-x.

47. Hussey, G.S., Goehring, L.S., Lunn, D.P., Hussey, S.B., Huang, T., Osterrieder, N., Powell, C., Hand, J., Holz, C., and Slater, J. (2013). Experimental infection with equine herpesvirus type 1 (EHV-1) induces chorioretinal lesions. Vet. Res. *44*, 118. https://doi.org/10.1186/1297-9716-44-118.

48. Guo, Y., Chen, J., Ji, W., Xu, L., Xie, Y., He, S., Lai, C., Hou, K., Li, Z., Chen, G., and Wu, Z. (2023). High-titer AAV disrupts cerebrovascular integrity and induces lymphocyte infiltration in adult mouse brain. Mol. Ther. Methods Clin. Dev. *31*, 101102. https://doi.org/10.1016/j.omtm.2023.08.021.

49. Ghoraba, H.H., Akhavanrezayat, A., Karaca, I., Yavari, N., Lajevardi, S., Hwang, J., Regenold, J., Matsumiya, W., Pham, B., Zaidi, M., et al. (2022). Ocular Gene Therapy: A Literature Review with Special Focus on Immune and Inflammatory Responses. Clin. Ophthalmol. *16*, 1753–1771. https://doi.org/10.2147/OPTH.S364200.

50. Reichel, F.F., Peters, T., Wilhelm, B., Biel, M., Ueffing, M., Wissinger, B., Bartz-Schmidt, K.U., Klein, R., Michalakis, S., and Fischer, M.D.; RD-CURE Consortium (2018). Humoral Immune Response After Intravitreal But Not After Subretinal AAV8 in Primates and Patients. Invest. Ophthalmol. Vis. Sci. *59*, 1910–1915. https://doi.org/10.1167/iovs.17-22494.

51. WB, B.J., Mehat, M.S., Venki, S., Robbie, S.J., Barker, S.E., Caterina, R., Anastasios, G., Mowat, F.M., Beattie, S.G., Gardner, P.J., et al. (2015). Long-Term Effect of Gene Therapy on Leber's Congenital Amaurosis. N. Engl. J. Med. *372*, 1887–1897. https://doi.org/10.1056/NEJMoa1414221.

52. Reichel, F.F., Dauletbekov, D.L., Klein, R., Peters, T., Ochakovski, G.A., Seitz, I.P., Wilhelm, B., Ueffing, M., Biel, M., Wissinger, B., et al. (2017). AAV8 Can Induce Innate and Adaptive Immune Response in the Primate Eye. Mol. Ther. *25*, 2648–2660. https://doi.org/10.1016/j.ymthe.2017.08.018.

53. Bouquet, C., Vignal Clermont, C., Galy, A., Fitoussi, S., Blouin, L., Munk, M.R., Valero, S., Meunier, S., Katz, B., Sahel, J.A., and Thomasson, N. (2019). Immune Response and Intraocular Inflammation in Patients With Leber Hereditary Optic Neuropathy Treated With Intravitreal Injection of Recombinant Adeno-Associated Virus 2 Carrying the ND4 Gene: A Secondary Analysis of a Phase 1/2 Clinical Trial. JAMA Ophthalmol. *137*, 399–406. https://doi.org/10.1001/jamaophthalmol.2018.6902.

54. Cukras, C., Wiley, H.E., Jeffrey, B.G., Sen, H.N., Turriff, A., Zeng, Y., Vijayasarathy, C., Marangoni, D., Ziccardi, L., Kjellstrom, S., et al. (2018). Retinal AAV8-RS1 Gene Therapy for X-Linked Retinoschisis: Initial Findings from a Phase I/IIa Trial by Intravitreal Delivery. Mol. Ther. *26*, 2282–2294. https://doi.org/10.1016/j.ymthe.2018.05.025.

55. Seitz, I.P., Michalakis, S., Wilhelm, B., Reichel, F.F., Ochakovski, G.A., Zrenner, E., Ueffing, M., Biel, M., Wissinger, B., Bartz-Schmidt, K.U., et al. (2017). Superior Retinal Gene Transfer and Biodistribution Profile of Subretinal Versus Intravitreal Delivery of AAV8 in Nonhuman Primates. Invest. Ophthalmol. Vis. Sci. *58*, 5792–5801. https://doi.org/10.1167/iovs.17-22473.

56. Aurnhammer, C., Haase, M., Muether, N., Hausl, M., Rauschhuber, C., Huber, I., Nitschko, H., Busch, U., Sing, A., Ehrhardt, A., and Baiker, A. (2012). Universal real-time PCR for the detection and quantification of adeno-associated virus serotype 2-derived inverted terminal repeat sequences. Hum. Gene Ther. Methods *23*, 18–28. https://doi.org/10.1089/hgtb.2011.034.

57. Eaton, J.S., Miller, P.E., Bentley, E., Thomasy, S.M., and Murphy, C.J. (2017). The SPOTS System: An Ocular Scoring System Optimized for Use in Modern Preclinical Drug Development and Toxicology. J. Ocul. Pharmacol. Therapeut. *33*, 718–734. https://doi.org/10.1089/jop.2017.0108.

58. Zhang, X., Anthony, B., Chai, Z., Lee Dobbins, A., Sutton, R.B., and Li, C. (2020). Membrane fusion FerA domains enhance adeno-associated virus vector transduction. Biomaterials *241*, 119906. https://doi.org/10.1016/j.biomaterials.2020.119906.

OMTM, Volume *32*

Supplemental information

Ocular toxicity, distribution, and shedding

of intravitreal AAV-eqIL-10 in horses

Kim Young, Tomoko Hasegawa, Naveen Vridhachalam, Nichol Henderson, Jacklyn H. Salmon, Trace F. McCall, Matthew L. Hirsch, and Brian C. Gilger

Table S1. Information on individual horses

Horse	Treatment group	Age	Gender	Breed	Body weight (kg)
1	Vehicle control (V)	11 years	Female	Thoroughbred	507
2	Low dose AAV8-eqIL10 (LD1)	12 years	Female	Pony	413
3	Low dose AAV8-eqIL10 (LD2)	13 years	Male (castrated)	Thoroughbred	569
4	High dose AAV8-eqIL10 (HD1)	20 years	Female	Quarter Horse	399
5	High dose AAV8-eqIL10 (HD2)	22 years	Female	Quarter Horse	499

Table S2. Neutralizing Antibody Testing.

Horse	Serum neutralizing antibodies	
	Prior to injection	Day 84 after injection
V	Not detected	Not detected
LD1	Not detected	Not detected
LD2	Not detected	4
HD1	Not detected	16
HD2	Not detected	16

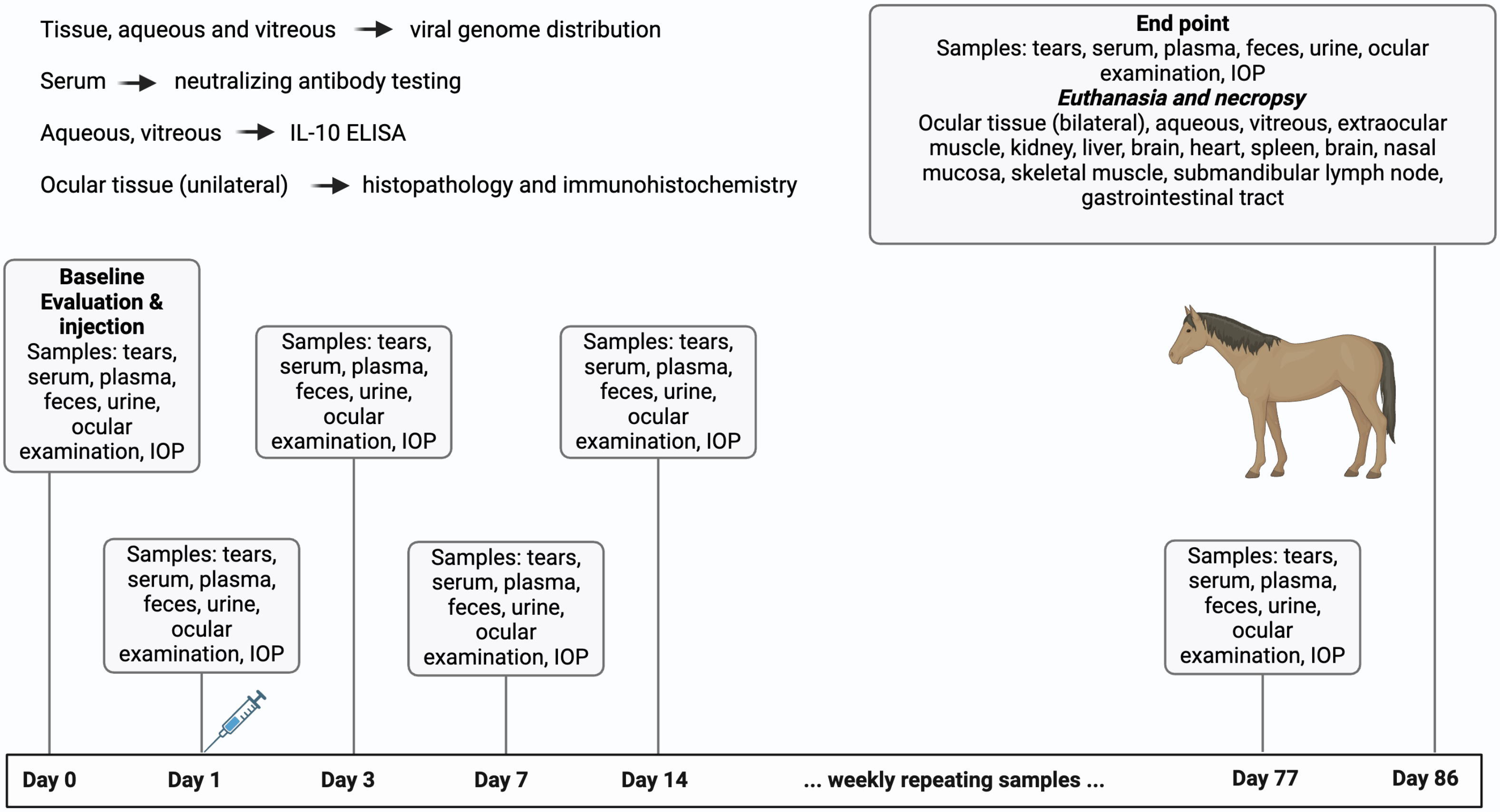

Figure S1. Sampling timeline and endpoint testing

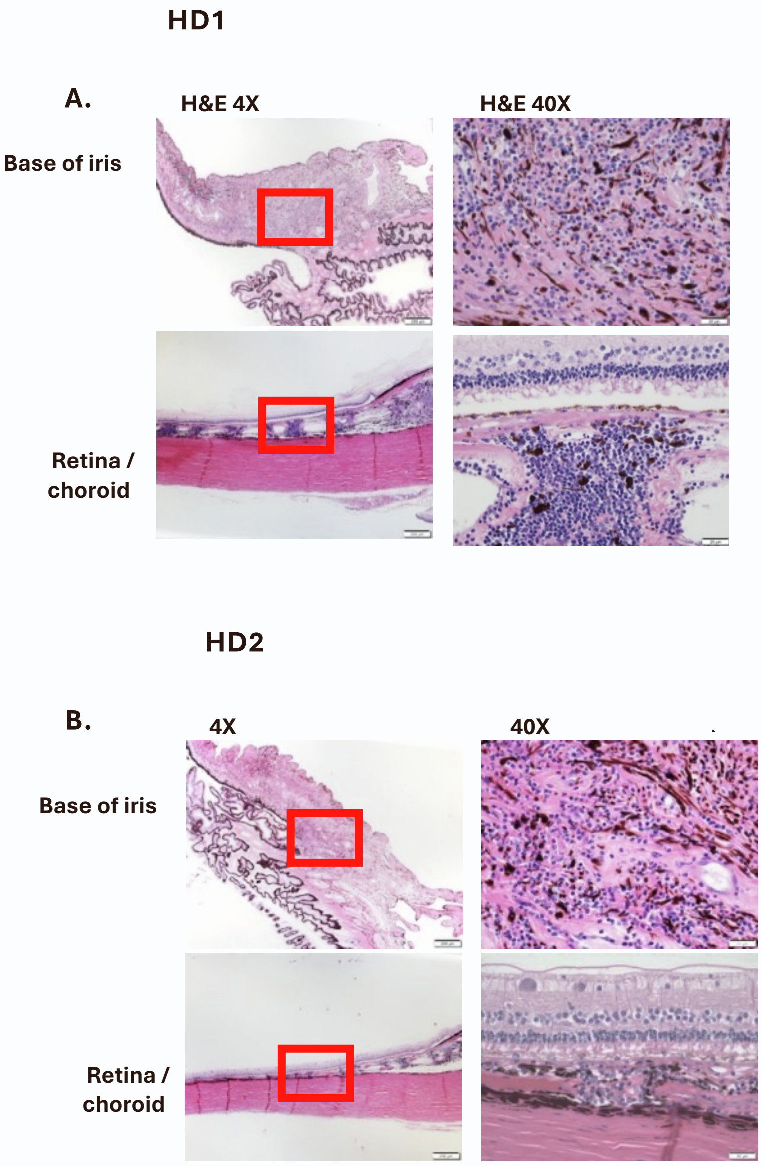

Figure S2 A. HD1. Ocular histology. In the iris and ciliary body (red box), there was diffuse infiltration of mononuclear cells in the iris and ciliary body (top row) and focal areas of mononuclear cells in the choroid (red box). **B.** HD2. Ocular histology. In the iris and ciliary body (red box), there was diffuse infiltration of mononuclear cells in the iris and ciliary body (top row) and focal areas of mononuclear cells in the choroid (red box).

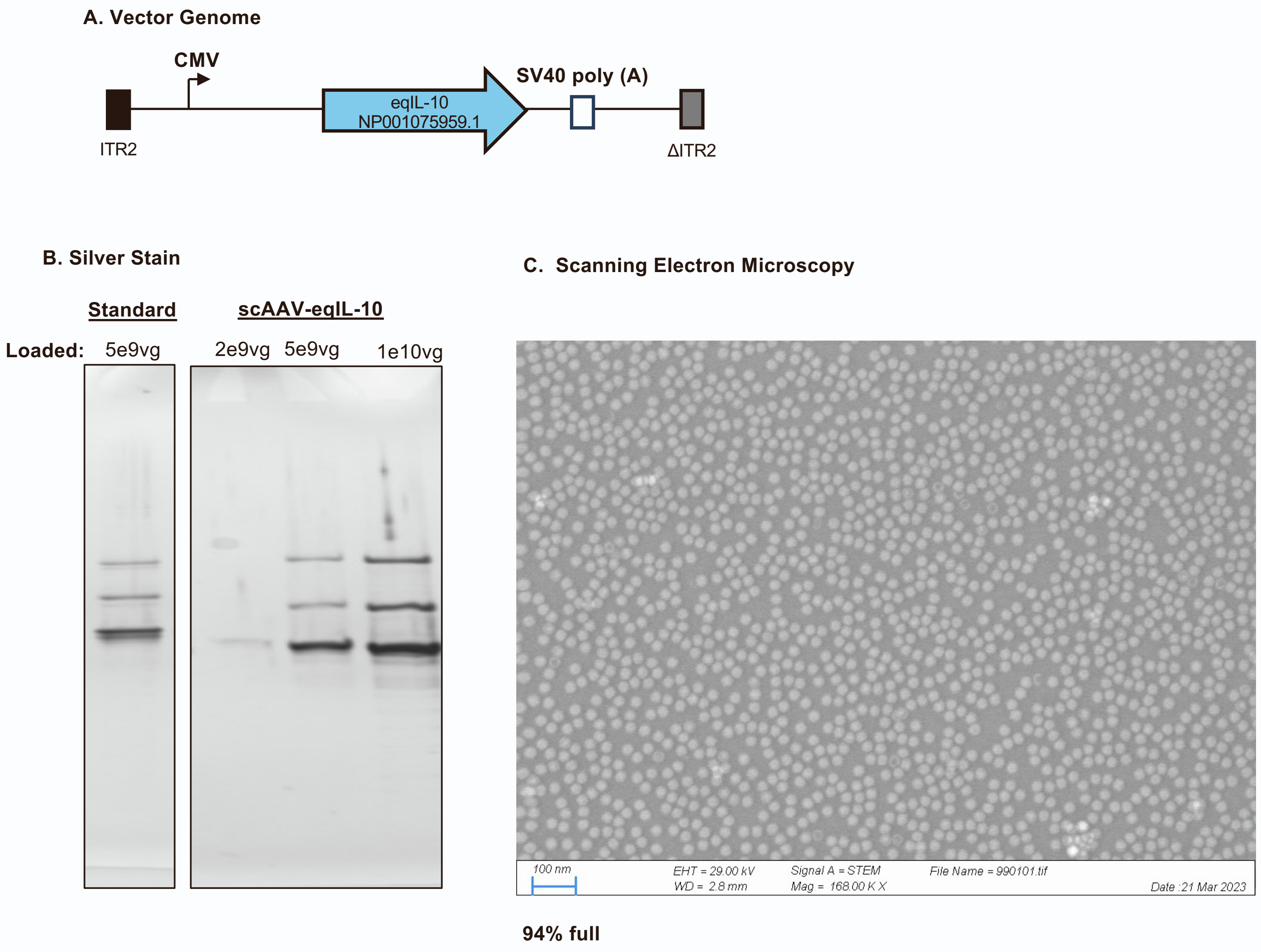

Figure S3. AAV8-eqIL-10 Characterization. **A.** A cartoon of the vector genome is depicted. **B.** Silver stain analysis revealed pure vector preparations at the indicated viral genomes. **C.** The full to empty capsid ratio of the preparation was >90% as demonstrated by electron microscopy.

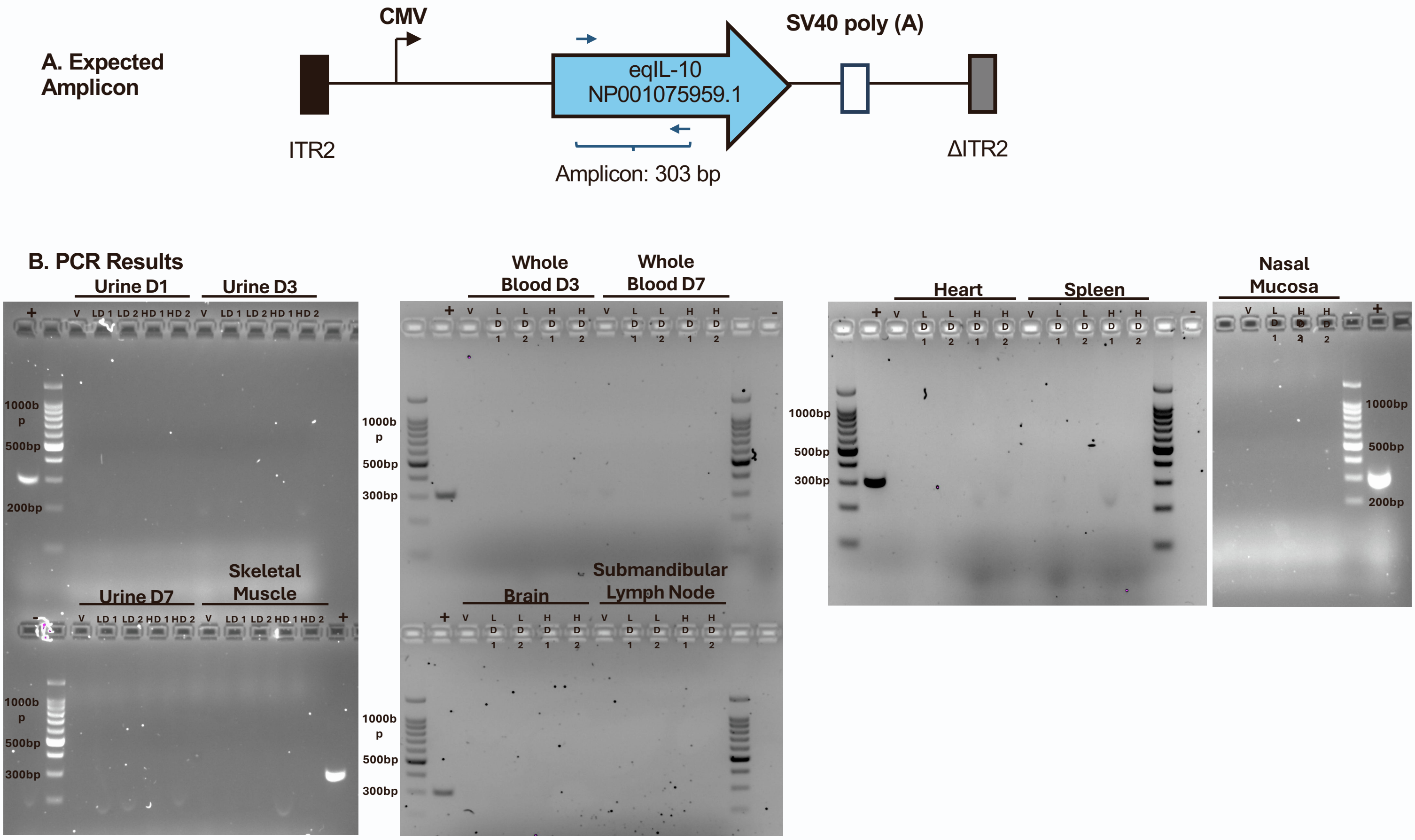

Figure S4. Vector Genome Biodistribution. The distribution of AAV8-eqIL-10 viral genomes in peripheral tissues was investigated via endpoint PCR. **A.** The forward and reverse primers annotated on the vector genome. The expected amplicon was 303 bp. **B.** The PCR amplicons were run on an agarose gel and visualized. All tissues from each horse did not have a 303 bp band, indicating that vector genomes were below the limit of detection in the indicated tissues. + = positive control; - = negative control; D = day; V = vehicle-treated; LD = low-dose treated; HD = high-dose treated.
